# Distinctions between the Koizumi and Zea Longa methods for middle cerebral artery occlusion (MCAO) model: a systematic review and meta-analysis of rodent data

**DOI:** 10.1038/s41598-023-37187-w

**Published:** 2023-06-23

**Authors:** Yong Li, Li Tan, Caixia Yang, Liying He, Lin Liu, Bowen Deng, Sijing Liu, Jinlin Guo

**Affiliations:** College of Pharmacy, Chengdu University of Traditional Chinese Medicine, Chengdu, China

**Keywords:** Diseases, Neurology, Pathogenesis

## Abstract

Ischemic stroke in rodents is usually induced by intraluminal middle cerebral artery occlusion (MCAO) via the common carotid artery plugging filament invented by Koizumi et al. (MCAO-KM), or the external carotid artery plugging filament created by Zea Longa et al. (MCAO-LG). A systematic review of the distinctions between them is currently lacking. Here, we performed a meta-analysis in terms of model establishment, cerebral blood flow (CBF), and cerebral ischemia–reperfusion injury (CIRI) between them, Weighted Mean Differences and Standardized Mean Difference were used to analyze the combined effects, Cochrane's Q test and the *I*^2^ statistic were applied to determine heterogeneity, sensitivity analysis and subgroup analysis were performed to explore the source of heterogeneity. Literature mining suggests that MCAO-KM brings shorter operation time (*p* = 0.007), higher probability of plugging filament (*p* < 0.001) and molding establishment (*p* = 0.006), lower possibility of subarachnoid hemorrhage (*p* = 0.02), larger infarct volume (*p* = 0.003), severer brain edema (*p* = 0.002), and neurological deficits (*p* = 0.03). Nevertheless, MCAO-LG shows a more adequate CBF after ischemia–reperfusion (*p* < 0.001), a higher model survival rate (*p* = 0.02), and a greater infarct rate (*p* = 0.007). In conclusion, the MCAO-KM method is simple to operate with a high modeling success rate, and is suitable for the study of brain edema under long-term hypoperfusion, while the MCAO-LG method is highly challenging for novices, and is suitable for the study of CIRI caused by complete ischemia–reperfusion. These findings are expected to benefit the selection of intraluminal filament MCAO models before undertaking ischemic stroke preclinical effectiveness trials.

## Introduction

Stroke is one of the leading causes of death and long-term disability across the world, a reliable stroke model is essential to produce an effective therapy for the destructive cerebrovascular accident^1–3^. Of note, about 87% of strokes in humans are ischemic strokes, and 70% of cerebral infarcts are caused by occlusion of the middle cerebral artery (MCA) and its branches^4^. The MCAO model conducted by intraluminal filament is considered as the most clinically relevant surgical model to mimic human ischemic stroke with the advantage of minor craniotomy trauma, stable controllability of reperfusion, as well as high reproducibility, etc^5,6^. This model was first reported by Jin-ichi Koizumi in 1986^7^, and modified by Enrique Zea Longa in 1989^8^, it has produced a profound influence on the research of ischemic stroke.

**Figure 1:**
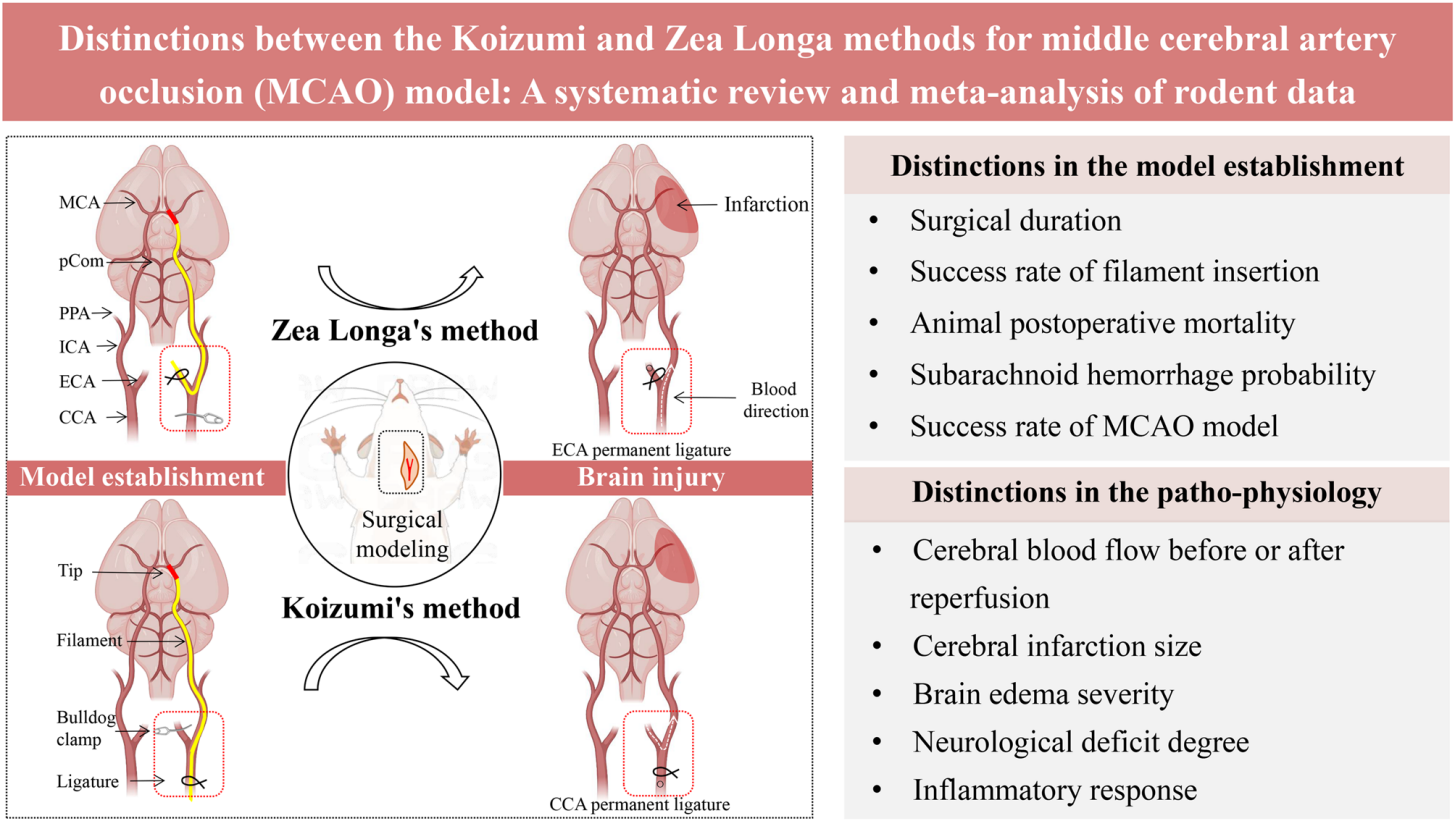
Distinctions in model establishment and patho-physiology between the Koizumi (MCAO-KM) and Zea Longa (MCAO-LG) methods for MCAO model. *MCA* middle cerebral artery, *pCom* posterior communicating artery, *PPA* pterygopalatine artery, *ICA* internal carotid artery, *ECA* external carotid artery, *CCA* common carotid artery.

The main distinctions between Koizumig's method (MCAO-KM) and Zea Longa's method (MCAO-LG) reside in the route of filament insertion to the cerebral artery and, subsequently, the CBF resupply degree (Fig. 1). Specifically, for MCAO-LG, the occlusion of the MCA by filament is accomplished via inserting the external carotid artery (ECA), and the ischemic tissue is reperfused by bilateral common carotid arteries (CCA). But, for MCAO-KM, the filament-blocked MCA is achieved by plugging through the CCA, and the reperfusion is accomplished via contralateral CCA using the circle of Willis^9^.

**Figure 2:**
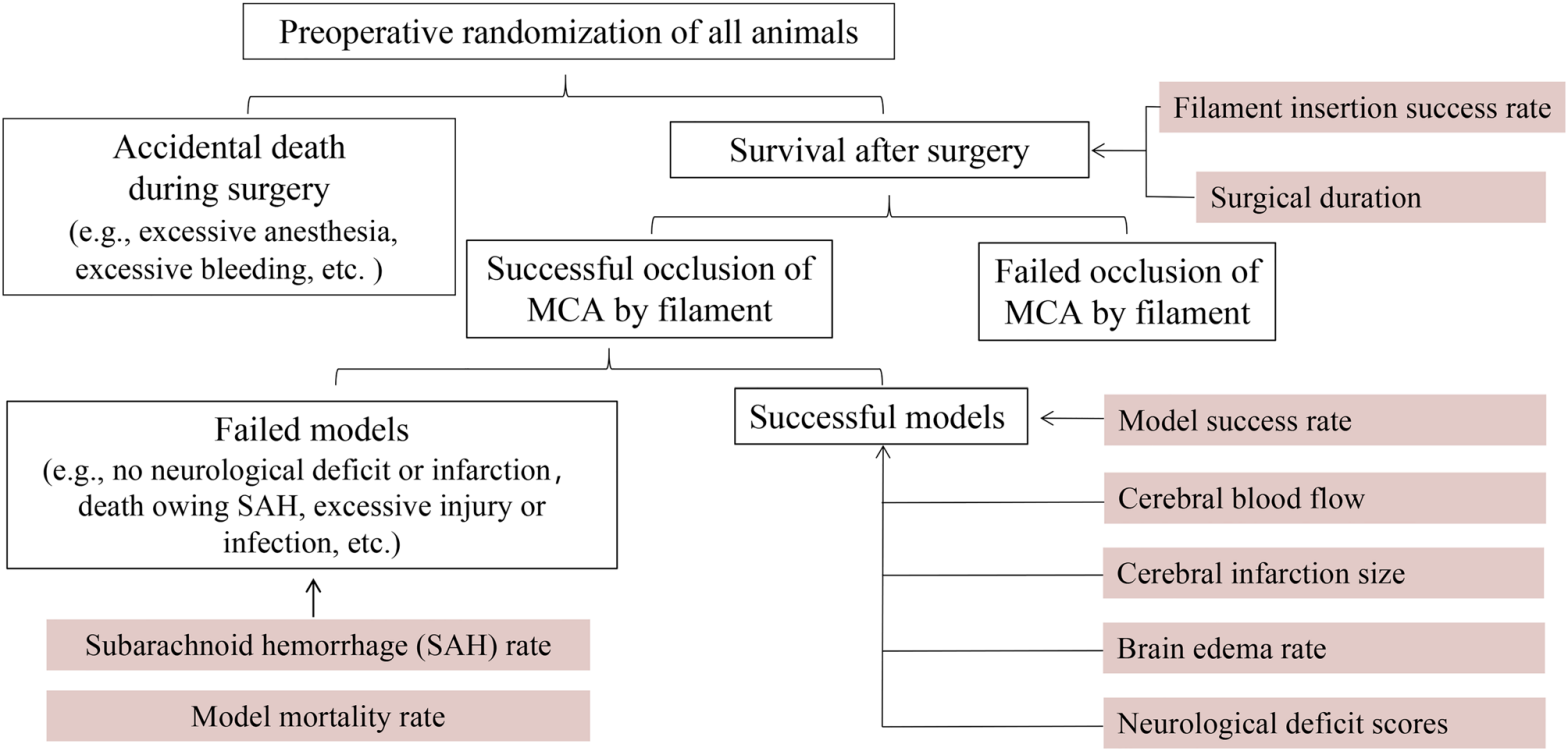
Experimental flowchart for MCAO modeling. The success rate of filament insertion = Animals with filament successfully inserted into the skull and blocked the MCA/animals involved in filament insertion; Postoperative mortality rate = Model animals that died after surgery (animals that died during surgery are not included)/model animals with successful insertion filament; Subarachnoid hemorrhage rate = Model animals with postoperative subarachnoid hemorrhage/model animals with successful insertion filament; MCAO model success rate = Successful model animals/animals involved in surgical modeling (successful model animal must meet one of the following characteristics: (1) TTC staining or MRI arise a significant infarct, or (2) neurological defect score (Zea Longa score) is 1 to 3 marks, and did not sacrifice during the test period).

The rodent intraluminal filament model is frequently employed in the research of preclinical neuroprotective medicines, however, there are numerous parameters influencing modeling performance, which is a significant obstacle for novices (Fig. 2). In addition, some scholars have conducted comparative studies on the two methods, but the results of previous studies were rooted in a relatively small sample, and there is no relevant meta-analysis to systematically compare their distinctions. Methodically reviewing and meta-analysis of all relevant studies in an objective and quantitative manner provide us with much credible and solid evidence to demonstrate the unique characteristics of each method. Therefore, in this study, we conducted a meta-analysis to determine the distinctions of modeling establishment and brain injury between the two methods, aiming to provide a reference for practitioners in this field.

## Methods

### Search strategy and study inclusion

The review was registered on the PROSPERO database before initiation (registration number CRD42022331652). A comprehensive search strategy was conducted in Web of Science, PubMed, Chinese National Knowledge Infrastructure (CNKI) and Wanfang database from their inceptions to July 2022 with the search terms: (Zea Longa's method OR external carotid artery insertion OR bilateral common carotid reperfusion) AND (Koizumi's method OR common carotid artery insertion OR unilateral common carotid reperfusion) AND (intraluminal filament model OR middle cerebral artery occlusion OR focal cerebral ischemia).

We included articles if (1) animal models: MCAO model was built by intraluminal filament method, without the restriction of ischemia and reperfusion duration; (2) interventions: the route of filament into the brain (CCA insertion or ECA insertion), or a surgical method (Koizumi's method or Zea Longa's method), or a reperfusion mode (bilateral common carotid reperfusion or unilateral common carotid reperfusion); (3) Outcomes: operation duration, inserting filament success rate, SAH probability, model animal mortality rate and modeling success rate, cerebral infarction size, brain edema rate, neurological deficit score (NDS).

### Data extraction and quality assessment

Two independent reviewers assessed related articles for eligibility, and extracted the following details: (1) document elements: first author and year of publication; (2) animal data: strains, sex, and weight (or age); (3) surgical details: anesthetic, ischemia duration, and reperfusion duration; (4) experimental outcomes: mean value, standard deviation, and sample size. WebPlotDigitizer (Version 4.4. 2020) was used for data extrapolation from graphs of published articles. In addition, any divergences were resolved by a senior author.

Quality of evidence in included studies was conducted based on a ten-item modified scale^10,11^: (a) peer-reviewed publication; (b) random allocation; (c) control of physiological parameter; (d) blinded conduct of the experiments; (e) blinded assessment of outcome; (f) use of anesthetic without significant neuroprotective activity; (g) co-morbidities (aged, diabetic and hypertensive etc.); (h) sample size calculation; (i) compliance with animal welfare regulations; (j) statement of potential conflict of interests. Among them, items (b), (c), (d), (e), (f), (g) and (h) may directly affect the model success rate.

### Statistical analysis

RevMan (version 5.4) was used for meta-analysis. Relative risk (RR) was estimated for all dichotomous variables. The weighted mean differences (WMD/MD) was calculated as a summary statistic if the continuous-type variable outcomes adopted the same scale, and the standardized mean difference (SMD) was used if the indexes were measured by various methods or techniques. Heterogeneity between studies was assessed via a standard chi-square test and *I*^2^ statistic, and *p* ≥ 0.01, *I*^2^ ≤ 50% and *p* < 0.01, *I*^2^ > 50% are considered low and high heterogeneity, respectively. If no statistical evidence of heterogeneity existed, the fixed effect (FE) model was performed with a 95% confidence interval (CI); if statistical heterogeneity was found, the sensitivity analysis and subgroup analysis were used. Statistical significance for all analyses was considered if *p* < 0.05. Egger’s tests were employed to detect publication bias, which was completed by Stata (version 15.1).

## Results

### Study search and selection

**Figure 3:**
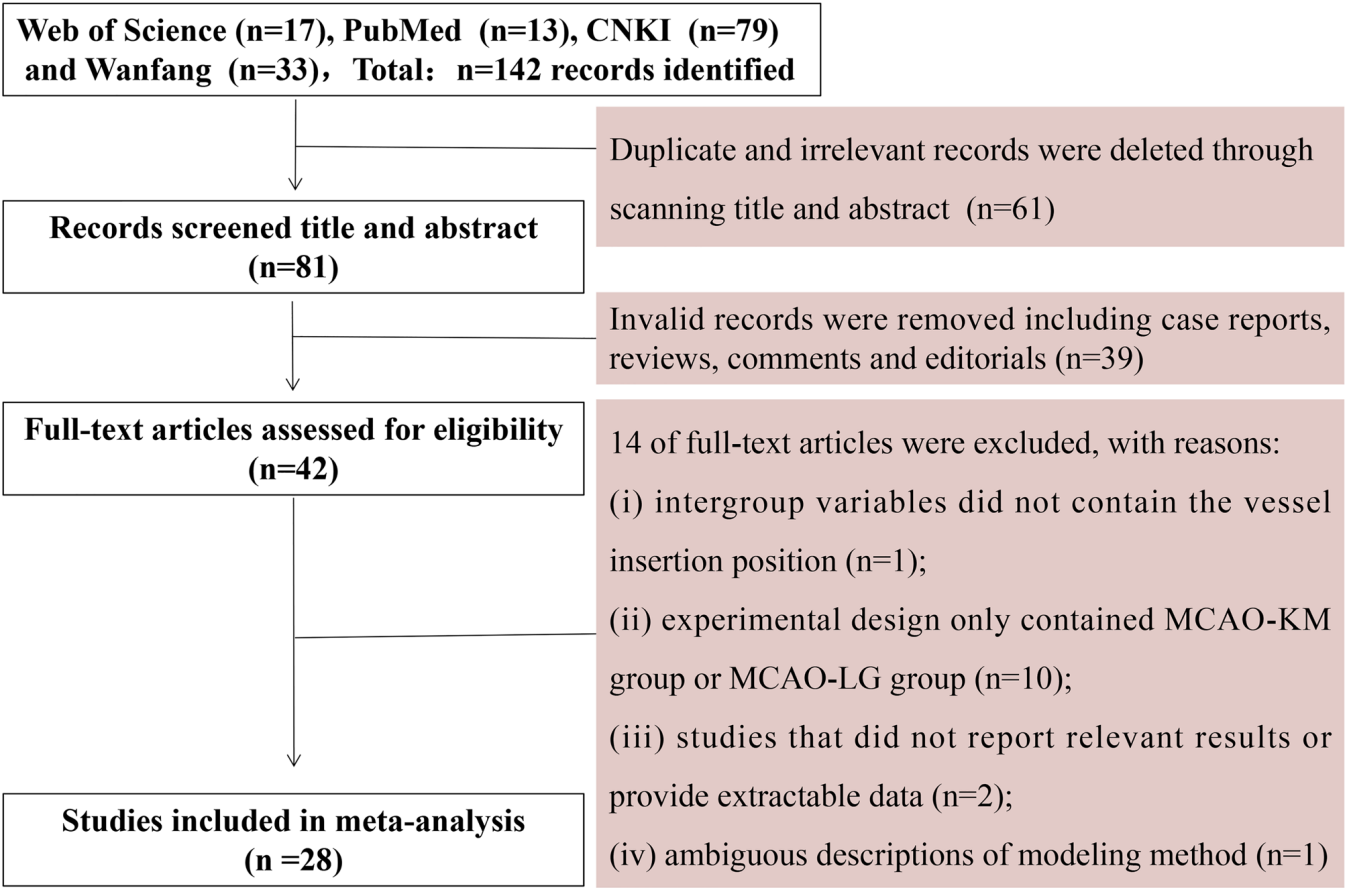
Flowchart of literature search.

After an initial search from English databases (Web of Science and PubMed) and Chinese databases (CNKI and Wanfang), a total of 142 potentially eligible studies were identified, and 61 studies were ruled out due to duplication and irrelevance. Further, thirty-nine studies were excluded owing to invalid records, and 14 studies were removed from the remaining 42 full-text articles post careful investigation. Ultimately, a total of 28 studies were included in the systematic meta-analysis (Fig. 3).

### Study characteristics

All the experimental animals were male-dominated rodents, except for one study sexes in half. Isoflurane was frequently used as anesthetic, others, including ketamine, pentobarbital sodium and fentanyl, etc. The treatment and plugging depth of the filament were reported in almost all literature. Cerebral ischemic injury in the included studies was induced by transient MCAO (tMCAO) or permanent MCAO (pMCAO). The characteristics of the included studies are provided in Table 1.

**Table 1:** Characteristics of the included study.

Study (Author, years)	Rodents	Anesthetic	Filament	Ischaemia time and hemisphere	Measurement indicators
Laing, R.J. 1993^16^	Wistar rats (no report, 300–400 g)	Fentanyl citrate + fluanisone + midazolam	1. Processing: tip coated with silicone (MCAO-KM)/tip burnt blunt round (MCAO-LG) 2. Diameter: no report 3. Insertion depth: 17–20 mm	No report, right	1. CBF 2. Success rate of filament insertion
Qu Qiumin 2000^34^	SD rats (male, 280–320 g)	Halothane + nitrous oxide	1. Processing: no report 2. Diameter: no report 3. Insertion depth: no report	2 h, left	1. Model success rate 2. Brain edema rate 3. Infarction rate
Cao Yongjun 2001^37^	Wistar rats (male, 200–250 g)	Chloral hydrate	1. Processing: tip burnt blunt round 2. Diameter: 4–0 nylon thread (0.2 mm) 3. Insertion depth: about 19.5–20.5 mm	6 h, right	1. rCBF 2. NDS (Zea Longa score) 3. Infarction size
Xi Gangming 2004 ^18^	Kunming mice (Male, 28–42 g)	Chloral hydrate	1. Processing: tip burnt blunt round and coated with polylysine 2. Diameter: 0.078–0.1 mm 3. Insertion depth: 8–10 mm	Permanent, right	1. Physiological parameters 2. Model mortality 3. Neurological scores 4. Infarction volume
Sun Guobing 2006^38^	SD rats (male, 250–300 g)	Chloral hydrate	1. Processing: tip burnt blunt round 2. Diameter: 4–0 nylon thread 3. Insertion depth: about 18 mm	2 h, right	1. Infarction rate 2. NF-κB expression
Johannes Woitzik 2006^35^	SD rats (male, 290–350 g)	Isoflurane	1. Processing: tip burnt blunt round (MCAO-LG) or coated with silicone (MCAO-KM) 2. Diameter: 5–0 polyamide filament (0.445–0.455 mm) 3. Insertion depth: 19 mm (based on CBF signals)	Permanent, right	1. Physiological parameters 2. CBF 3. Infarction rate 4. Brain edema rate 5. NDS
Hiroaki Sakai 2007^40^	Wistar rats (male, 10 weeks-age)	Isoflurane	1. Processing: tip coated with silicone 2. Diameter: 0.38 mm 3. Insertion depth: 19–20 mm	50 min, right	1. Infarction volume 2. NDS
Cam Ertugrul 2008^19^	Wistar rats (male, 285–370 g)	Isoflurane	1. Processing: abrading the tip with sandpaper 2. Diameter: 0.23 mm 3. Insertion depth: 18 mm	Permanent, left	1. Physiological parameters 2. CBF 3. Model mortality 4. Model success rate 5. Subarachnoid hemorrhage rate 6. Infarction volume
Sun Yu 2008^12^	SD rats (male, 240–280 g)	Chloral hydrate	1. Processing: tip burnt blunt round 2. Diameter: 4–0 filaments (0.22 mm) 3. Insertion depth: 19–20 mm	2 h, right	1. Operation duration 2. Model success rate 3. Model mortality 4. Infarction rate 5. NDS (Zea Longa score) 6. HE staining
Xiang Heng 2008^20^	SD rats (male, 300–350 g)	Chloral hydrate	1. Processing: tip coated with silicone 2. Diameter: 0.26–0.30 mm 3. Insertion depth: a feel of slight resistance	Permanent, left	1. Model success rate 2. Model mortality 3. Infarction rate 4. NDS (Zea Longa score)
Yang Zanzhang 2008^21^	Wistar rats (sex in half, 260–300 g)	Pentobarbital sodium	1. Processing: tip burnt blunt round 2. Diameter: fishing thread (0.235 mm) 3. Insertion depth: about 18 mm (a feel of slight resistance)	Permanent, left	1. Model success rate 2. Infarction rate 3. NDS (Zea Longa score)
Yang Debing 2009^17^	Wistar rats (male, 230–280 g)	No report	1. Processing: no report 2. Diameter: no report 3. Insertion depth: a feel of slight resistance	2 h, right	1. Model success rate 2. Model mortality 3. NDS (Zea Longa score)
Trueman, R, C 2011^22^	Wistar rats (male, 10–12 weeks-age)	Isoflurane	1. Processing: tip coated with silicone 2. Diameter: 0.39–0.41 μm 3. Insertion depth: depending on changes of CBF	1 h, right	1. Model success rate 2. Model mortality 3. CBF 4. Infarction rate 5. Body weight change 6. Behavioral testing 7. Survival rate of neurons
Liu Jianren 2012^23^	SD rats (male, 200–250 g)	Chloral hydrate	1. Processing: tip coated with silicone 2. Diameter: no report 3. Insertion depth: no report	1.5 h, right	1. Physiological parameters 2. CBF 3. Infarction volume 4. Brain edema rate 5. NDS (Berdeson/Garcia score)
Zhao Kai 2012^32^	Wistar rats (male, 280–300 g)	Chloral hydrate	1. Processing: tip burnt blunt round or cut into a flat end 2. Diameter: 0.26 mm 3.insertion depth: 18–20 mm (a feel of slight resistance)	100 min, right	1. Model success rate 2. NDS (Zea Longa score) 3. Infarction rate 4. HE staining
Tang Qiqiang 2013^33^	SD rats (male, 250–300 g)	Chloral hydrate	1. Processing: tip coated with silicone 2. Diameter: 0.21–0.22 mm or 0.27–0.28 mm or 0.28–0.29 mm; 3. Insertion depth: 16–20 mm	Permanent, right	1. Physiological parameters 2. Success rate of filament insertion 3. NDS (Zea Longa score) 4. Infarction rate 5. Brain edema rate 6. HE staining
Fan Ruijuan 2014^14^	SD rats (male, 250–300 g)	Chloral hydrate	1. Processing: tip coated with polylysine 2. Diameter: 0.34–0.38 mm 3. Insertion depth: 19–20 mm (A feel of slight resistance)	Transient (no ischaemia time), right	1. Operation duration 2. Success rate of filament insertion 3. Model success rate 4. Model mortality 5. Subarachnoid hemorrhage rate
Morris, G.P. 2015^15^	C57BL/6 mice (male, 8–13 weeks-age)	Ketamine + xylazine	1. Processing: silicone coated for 9–10 mm (MCAO-KM)) or 2–3 mm (MCAO-LG) 2. Diameter: 0.19–0.23 mm 3. Insertion depth: depending on changes of CBF	1 h, right	1. CBF 2. Infarction rate 3. Body weight change
Smith, H.K. 2015^24^	C57BL/6 mice (male, 25–29 g)	Ketamine/xylazine	1. Processing: tip coated with silicone 2. Diameter: 0.18 mm 3. Insertion depth: a feel of slight resistance	0.5 h, no report	1. CBF 2. Model mortality 3. Infarction rate 4. NDS (mNSS score) 5. Inflammation
Zheng Jianfeng 2016^25^	SD rats (male, 280–320 g)	Pentobarbital sodium	1. Processing: tip coated with polylysine 2. Diameter: 0.26–0.28 mm 3. Insertion depth: 18–20 mm	2 h/permanent, right	1. Model mortality 2. Infarction rate 3. NDS (Zea Longa score)
Zuo Xialin 2013^26^	SD rats (male, 250–280 g)	Chloral hydrate	1. Processing: tip coated with solid paraffin; 2. Diameter: 0.28 mm 3. Insertion depth: about 18 mm	1 h, left	1. Model mortality 2. Infarction rate 3. BBB integrity 4. NDS (Zea Longa score) 5. BDNF expression
Cai Qiang 2016^13^	C57BL/6 mice (male, 20–25 g)	Isoflurane	1. Processing: tip coated with silicone 2. Diameter: 0.21–0.22 mm 3. Insertion depth: a feel of slight resistance	1 h, right	1. CBF 2. Operation duration 3. Model mortality 4. Infarction rate 5. NDS (Zea Longa score)
Melissa, T.L. 2017^36^	C57BL/6 mice (male, 24–31 g)	Isoflurane	1. Processing: tip coated with silicone 2. Diameter: 0.18–0.20 mm 3. Insertion depth: depending on changes of CBF	1 h, right	1. rCBF 2. Infarction volume
Wang Dongliang 2019^27^	C57BL/6 mice (male, 20–22 g)	Isoflurane	1. Processing: tip coated with silicone 2. Diameter: no report 3. Insertion depth: 8–12 mm or a feel of slight resistance	1 h, right	1. Model mortality 2. Model success rate 3. Infarction rate 4. Brain water content 5. Neuronal apoptosis rate
Onufriev, M.V. 2021^28^	Wistar rats (male, 200–300 g)	Isoflurane	1. Processing: tip burnt blunt round 2. Diameter: 3–0 nylon monofilament 3. Insertion depth: no report	1 h, left	1. Model mortality 2. NDS 3. Infarction rate 4. Inflammation
Wang Wenxiu 2022^29^	SD rats (male, 250–300 g)	Chloral hydrate	1. Processing: tip burnt blunt round and coated with polylysine 2. Diameter: 0.34–0.38 mm 3. Insertion depth: 21–22 mm	2 h, right	1. Success rate of filament insertion 2. Model mortality 3. NDS (Zea Longa score)
Yang Zhong 2022^30^	C57Bl6 mice (male, 8–10 weeks-age)	Isoflurane	1. Processing: no report 2. Diameter: no report 3. Insertion depth: no report	1.5 h, right	1. CBF 2. NDS 3. Infarction volume 4. Body weight change 5. Inflammation 6. Neuronal apoptosis rate
Helena, J. 2022^31^	C57Bl6 mice (male, 3–6 months old)	Isoflurane	1. Processing: tip coated with silicone 2. Diameter: 0.19–0.21 mm 3. Insertion depth: depending on changes of CBF	0.5 h, left	1. CBF 2. Infarction volume 3. Body weight change 4. NDS

*BDNF* brain derived neurotrophic factor, *CBF* cerebral blood flow, *NDS* neurological deficit score, *BBB* blood–brain barrier, *HE staining* hematoxylin and eosin staining.

The comparison of model establishment, CBF and ischemic brain injury was the focus of our study. Among them, the success rate of plugging filament, incidence of SAH, model mortality rate and model success rate were analyzed as dichotomous variables. While, the surgical operation duration, cerebral infarction size, brain edema rate and neurological deficit score were analyzed as continuous variables.

### Quality of included studies

Study quality scores for 28 studies counted in this meta-analysis were summarized in Table 2. The quality scores varied from 3 to 8 with an average of 5.46. All the studies were peer-reviewed publications. Twenty-four studies declared randomization of group allocation, and 21 studies described the monitoring of physiological parameters. Besides, one study masked the details of experimental designs, and 14 studies reported a blinded assessment of the outcome. Thirteen studies avoided the use of anesthetics with marked neuroprotective properties. None of the studies reported the application of co-morbidities in animals. Twenty-five studies reported sample size calculation. Among all of them, 18 studies stated compliance with animal welfare regulations, and 9 studies addressed the conflict of interests.

**Table 2:** Quality evaluation of included studies.

Year	Lead author	A	B	C	D	E	F	G	H	I	J	Total
1993	Laing, R.J.^16^	+		+					+			3
2000	Qu Qiumin^34^	+	+						+			3
2001	Cao Yongjun^37^	+	+	+			+		+			5
2004	Xi Gangming^18^	+	+	+		+			+			5
2006	Sun Guobing^38^	+	+				+		+			4
2006	Johannes Woitzik^35^	+	+	+		+				+	+	6
2007	Hiroaki Sakai^40^	+	+	+		+			+	+		6
2008	Cam Ertugrul^19^	+		+			+		+	+		5
2008	Sun Yu^12^	+	+				+		+			4
2008	Xiang Heng^20^	+	+				+		+	+		5
2008	Yang Zanzhang^21^	+	+	+			+		+			5
2009	Yang Debing^17^	+	+						+			3
2011	Trueman, R, C.^22^	+	+	+	+	+			+	+		7
2012	Liu Jianren^23^	+	+	+		+	+		+	+	+	8
2012	Zhao Kai^32^	+	+				+		+			4
2013	Tang Qiqiang^33^	+	+	+			+		+	+	+	7
2014	Fan Ruijuan^14^	+	+			+	+		+			5
2015	Morris, G.P.^15^	+	+	+		+			+	+	+	7
2015	Smith, H.K.^24^	+		+		+				+		4
2016	Zheng Jianfeng^25^	+	+	+			+		+	+		6
2016	Zuo Xialin^26^	+	+	+		+	+		+	+		7
2016	Cai Qiang^13^	+		+		+				+	+	5
2017	Melissa, T.L.^36^	+	+	+		+			+	+	+	7
2019	Wang Dongliang^27^	+	+	+					+	+		5
2021	Onufriev, M.V.^28^	+	+	+		+			+	+	+	7
2022	Wang Wenxiu^29^	+	+	+			+		+	+		6
2022	Yang Zhong^30^	+	+	+		+			+	+	+	7
2022	Helena, Justic^31^	+	+	+		+			+	+	+	7

Study quality items are A, Peer-reviewed publication; B, Random grouping; C, Monitoring of physiological parameters (e.g. temperature, blood pressure, gases); D, Blinded conduct of ischemia; E, Blinded assessment of outcomes; F, Use of anesthetic without marked intrinsic neuroprotective properties (e.g., isoflurane, ketamine, halothane); G, Animal with co-morbidities (e.g., diabetic, aged, or hypertensive); H, Sample size calculation; I, Statement of compliance with animal welfare regulations; J, Statement of potential conflict of interests.

### Comparisons between the two methods in model establishment

#### Distinction on operation duration

**Figure 4:**
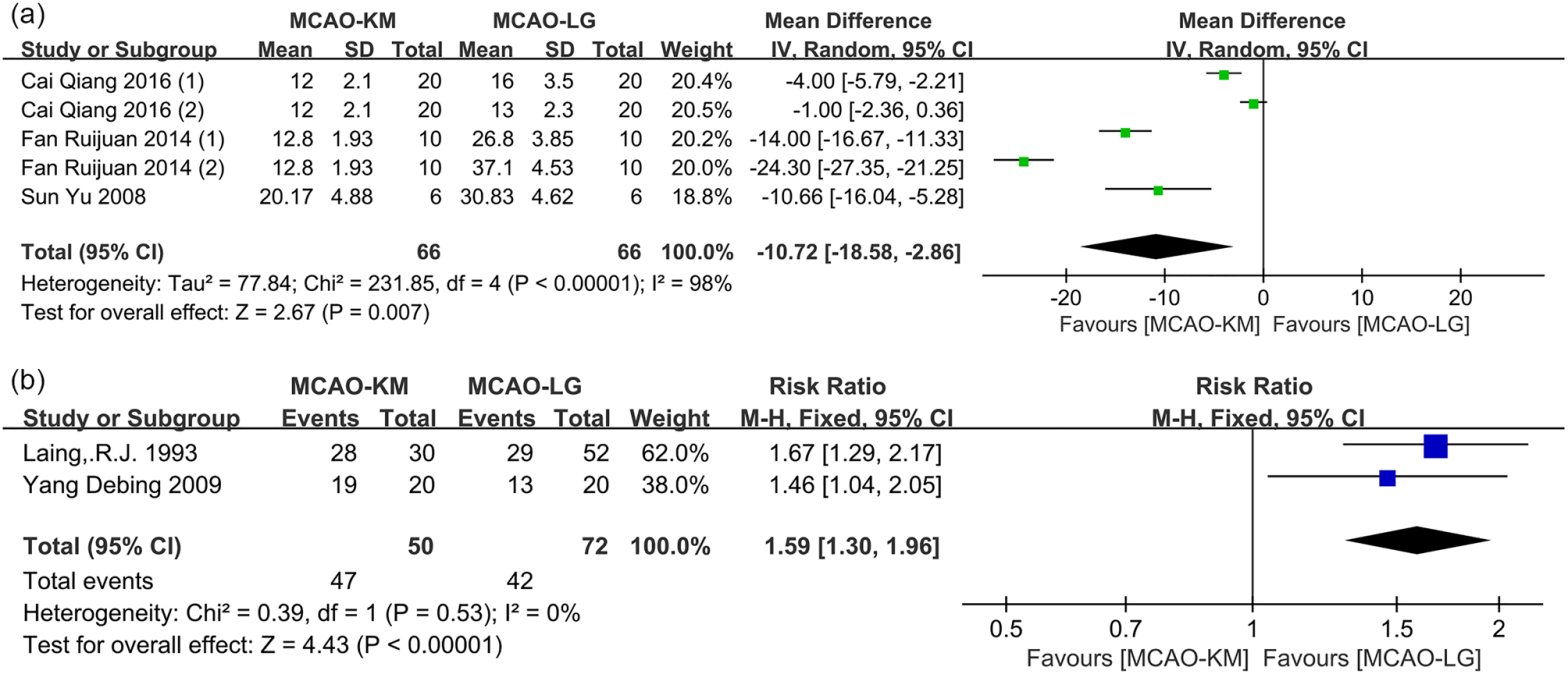
Comparison of (**a**) operation duration and (**b**) success rate of filament insertion between the MCAO-KM and MCAO-LG.

Four studies^12–15^ reported the duration of surgical operation, and one^15^ was excluded due to the absence of an extractable date. Overall, the operation duration of MCAO-KM was significantly shorter than that of MCAO-LG (MD = − 10.72, 95% CI [− 18.58, − 2.86], *p* = 0.007, heterogeneity* I*^2^ = 98%, *p* < 0.00001) (Fig. 4a). The separation and ligation of the pterygopalatine artery (PPA), as well as the surgical proficiency, may be important sources of heterogeneity. Of note, the study^15^ also pointed out that the operation time of MCAO-KM is shorter than MCAO-LG, which is consistent with the pooled MD estimation.

#### Distinction on probability of plugging filament

The success rates of filament insertion were reported in three studies^14,16,17^. Overall, the MCAO-KM shows a higher probability of plugging filament than MCAO-LG (RR = 1.43, 95% CI [1.20, 1.70], *p* < 0.0001, heterogeneity *I*^2^ = 84%, *p* = 0.002) (Supplementary Fig. 1), and one study^14^ should be the source of heterogeneity through sensitivity analyses. After the removal of this study, the pooled result between the two methods remained similar and the heterogeneity was decreased (RR = 1.59, 95% CI [1.30, 1.96], *p* < 0.00001, heterogeneity *I*^2^ = 0%, *p* = 0.53) (Fig. 4b).

#### Distinction on postoperative mortality

**Figure 5:**
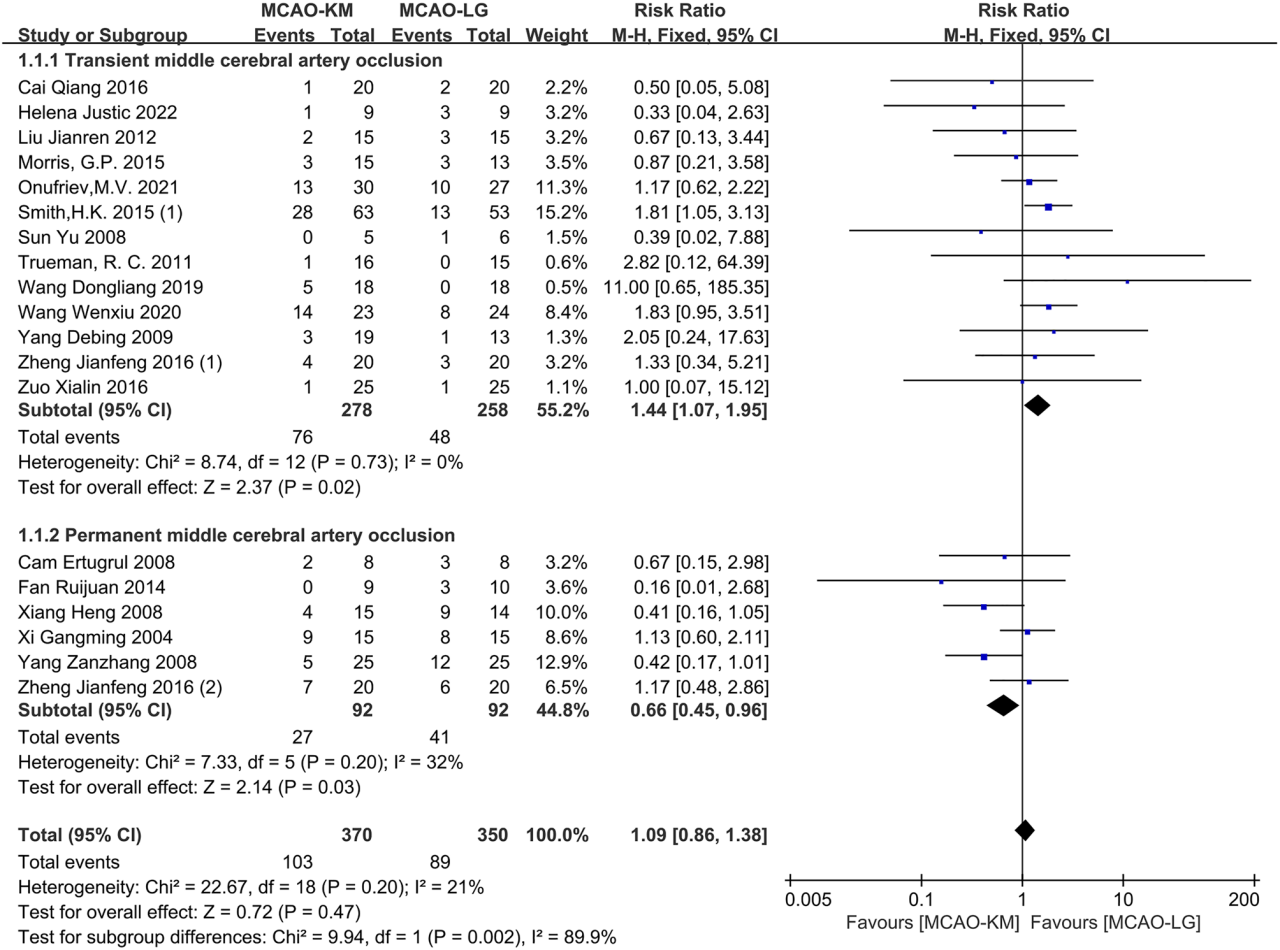
Comparison of postoperative mortality between MCAO-KM and MCAO-LG.

Nineteen studies^12–15,17–31^ reported rodent mortality after plugging filament, and one study^30^ was excluded due to a lack of extractable data. Overall, and, no significant difference between the two methods was observed (RR = 1.09, 95% CI [0.86, 1.38], p = 0.47, heterogeneity *I*^2^ = 21%, p = 0.20) (Fig. 5). Of note, sub-analyses show that modeling animals induced by MCAO-KM exhibited higher mortality than that by MCAO-LG in the tMCAO subgroup (RR = 1.44, 95% CI [1.07, 1.95], *p* = 0.02, heterogeneity *I*^2^ = 0%, *p* = 0.73), whereas, the opposite result was found in the pMCAO subgroup (RR = 0.66, 95% CI [0.45, 0.96], *p* = 0.03, heterogeneity *I*^2^ = 32%, *p* = 0.20). The *p*-value of Egger's regression test in the tMCAO subgroup was 0.291, indicating no significant publication bias (Supplementary Fig. 2).

#### Distinction on occurence of SAH

**Figure 6:**
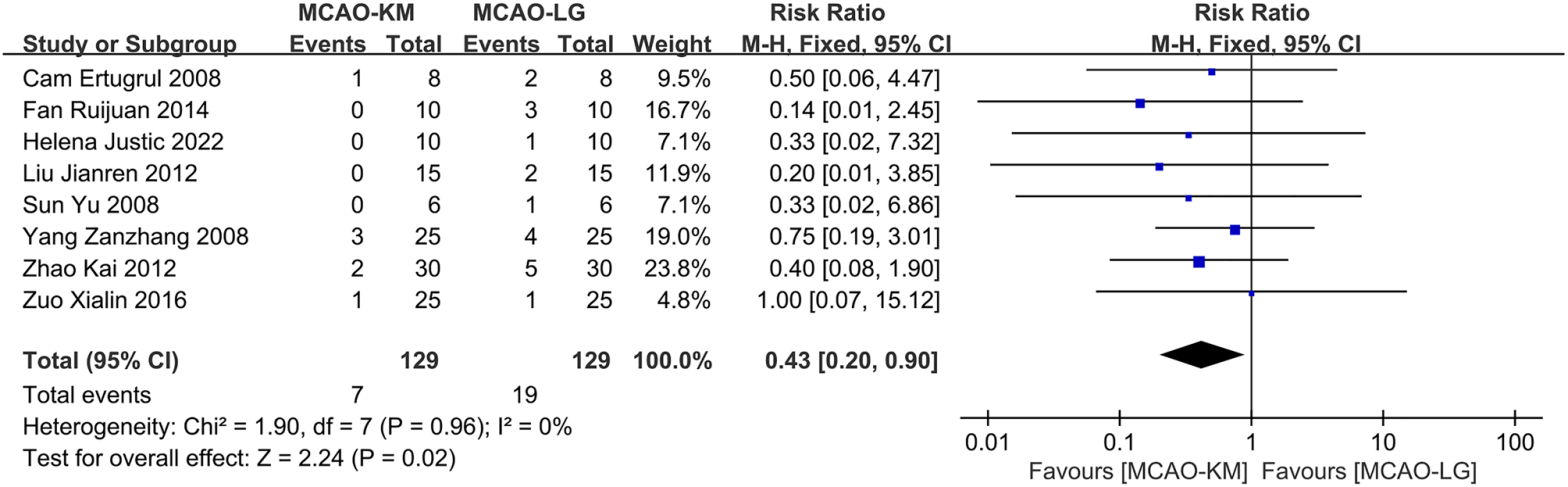
Comparison of postoperative SAH between MCAO-KM and MCAO-LG.

Nine studies^12,14,19,21,23,26,31–33^ reported the occurrence of SAH after the operation, and one study^33^ was excluded due to a lack of detailed data. Overall, the meta-analysis results indicate that the modeling method of MCAO-KM causes a lower probability of SAH compared to the MCAO-LG with no substantial heterogeneity (RR = 0.43, 95% CI [0.20, 0.90], *p* = 0.02, heterogeneity *I*^2^ = 0%, *p* = 0.96) (Fig. 6).

#### Distinction on success rate of MCAO models

The success rates of MCAO models were assessed in 16 studies^12,14,15,17,19–23,25,27–29,32–34^. Overall, the MCAO-KM brings a statistically higher probability of model success than the MCAO-LG (RR = 1.14, 95% CI [1.03, 1.27], *p* = 0.01, heterogeneity *I*^2^ = 45%, *p* = 0.02). Subgroup analyses suggest that the MCAO-KM supports higher model success rate in the pMCAO subgroup (RR = 1.34, 95% CI [1.09, 1.66], *p* = 0.006, heterogeneity *I*^2^ = 0%, *p* = 0.51), but not tMCAO (RR = 1.06, 95% CI [0.94, 1.20], *p* = 0.33, heterogeneity *I*^2^ = 46%, *p* = 0.04) (Supplementary Fig. 3a).

**Figure 7:**
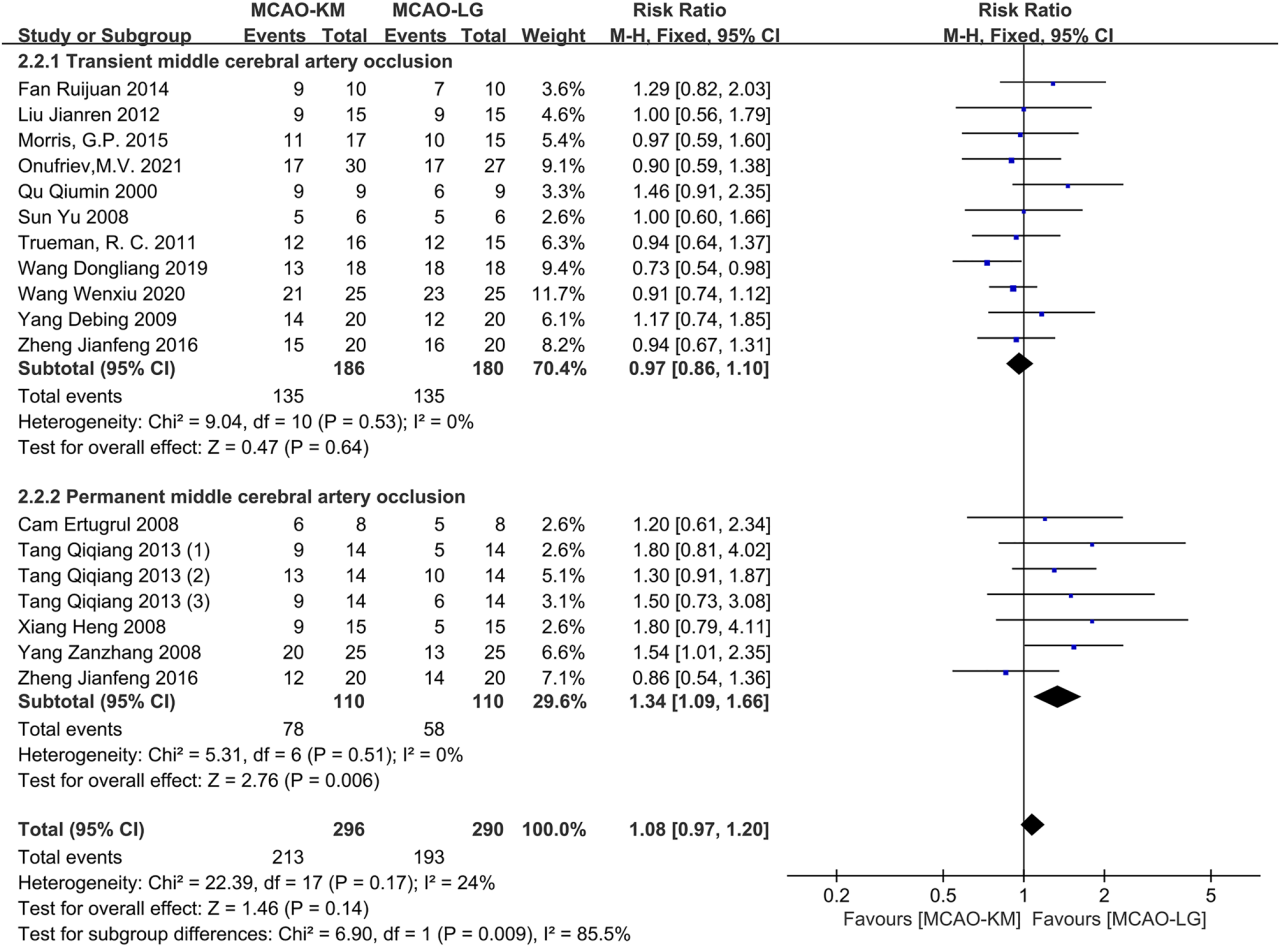
Comparison of modeling success rates between MCAO-KM and MCAO-LG.

Sensitivity analysis of the tMCAO subgroups revealed one study^32^ may be the source of heterogeneity. After deletion of this study, no significant difference between the two methods was found in overall effect (RR = 1.08, 95% CI [0.97, 1.20], *p* = 0.14, heterogeneity *I*^2^ = 24%, *p* = 0.17) as well as tMCAO subgroup (RR = 0.97, 95% CI [0.86, 1.10], *p* = 0.64, heterogeneity *I*^2^ = 0%, *p* = 0.53). However, the MCAO-KM method's success rate remains higher in the pMCAO subgroup (Fig. 7). The *p*-value of Egger's regression test in the tMCAO subgroup was 0.089, indicating no significant publication bias (Supplementary Fig. 3b,c).

### Comparisons between the two methods in CBF and brain injury

#### Distinction on CBF after ischemia or ischemia–reperfusion

**Figure 8:**
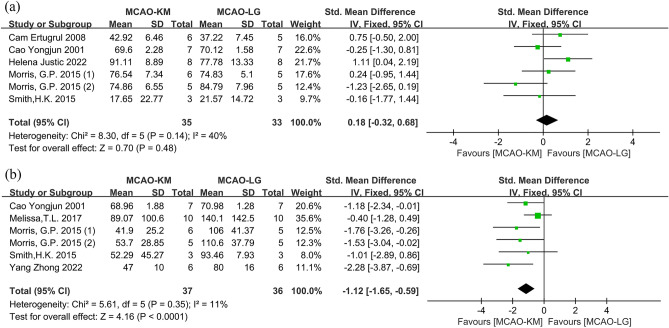
Comparison of CBF between MCAO-KM and MCAO-LG after (**a**) cerebral ischemia and (**b**) ischemia–reperfusion.

Eight studies^13,15,24,30,31,35–37^ with 9 comparisons were applied to evaluate CBF after cerebral ischemia or ischemia–reperfusion between MCAO-KM and MCAO-LG, and one study ^13^ was excluded due to lack of sample size, and another study^35^ was also excluded because it did not reperfuse by removing the filament (Table 3). Overall, there was no significant difference in CBF between the two methods during ischemia (SMD = 0.18; 95% CI [− 0.32, 0.68], *p* = 0.48; heterogeneity *I*^2^ = 40%, *p* = 0.14) (Fig. 8a). Interestingly, after reperfusion, the CBF achieved by MCAO-KM was dramatically lower than that by MCAO-LG (SMD = − 1.34; 95% CI [− 1.85, − 0.83], *p* < 0.00001; heterogeneity *I*^2^ = 60%, *p* = 0.02) (Supplementary Fig. 4). After the exclusion of one study^31^ as a potential source of heterogeneity, the CBF outcomes between the two methods remained similar and the heterogeneity was significantly reduced (SMD = − 1.12; 95% CI [− 1.65, − 0.59], *p* < 0.0001; heterogeneity *I*^2^ = 11%, *p* = 0.35) (Fig. 8b).

**Table 3:** Details of cerebral blood flow test.

Lead author	Year	Test method	Ischemia duration	Test time
Cai Qiang^13^	2016	Laser Doppler flowmetry	1 h	5 min after reperfusion
Cam Ertugrul^19^	2008	Laser Doppler flowmetry	Permanent	20 min after occlusion
Cao Yongjun^37^	2001	Hydrogen clearance method	6 h	0.5 h/1 h/2 h after reperfusion
Melissa, T.L.^36^	2017	Laser Doppler flowmetry	1 h	5 min after reperfusion
Helena Justic^31^	2022	Laser Doppler flowmetry	0.5 h	No report
Johannes Woitzik^35^	2006	Laser Doppler flowmetry	Permanent	240 min after occlusion
Morris, G.P.^15^ (thin filament)	2015	Laser Doppler flowmetry	1 h	4 h after reperfusion
Morris,.G.P.^15^ (thick filament)	2016	Laser Doppler flowmetry	1 h	4 h after reperfusion
Smith, H.K.^24^	2015	Laser Doppler flowmetry	0.5 h	5 min after reperfusion
Yang Zhong^30^	2022	Laser Doppler flowmetry	1.5 h	10 min after reperfusion

#### Distinction on cerebral infarction size

**Figure 9:**
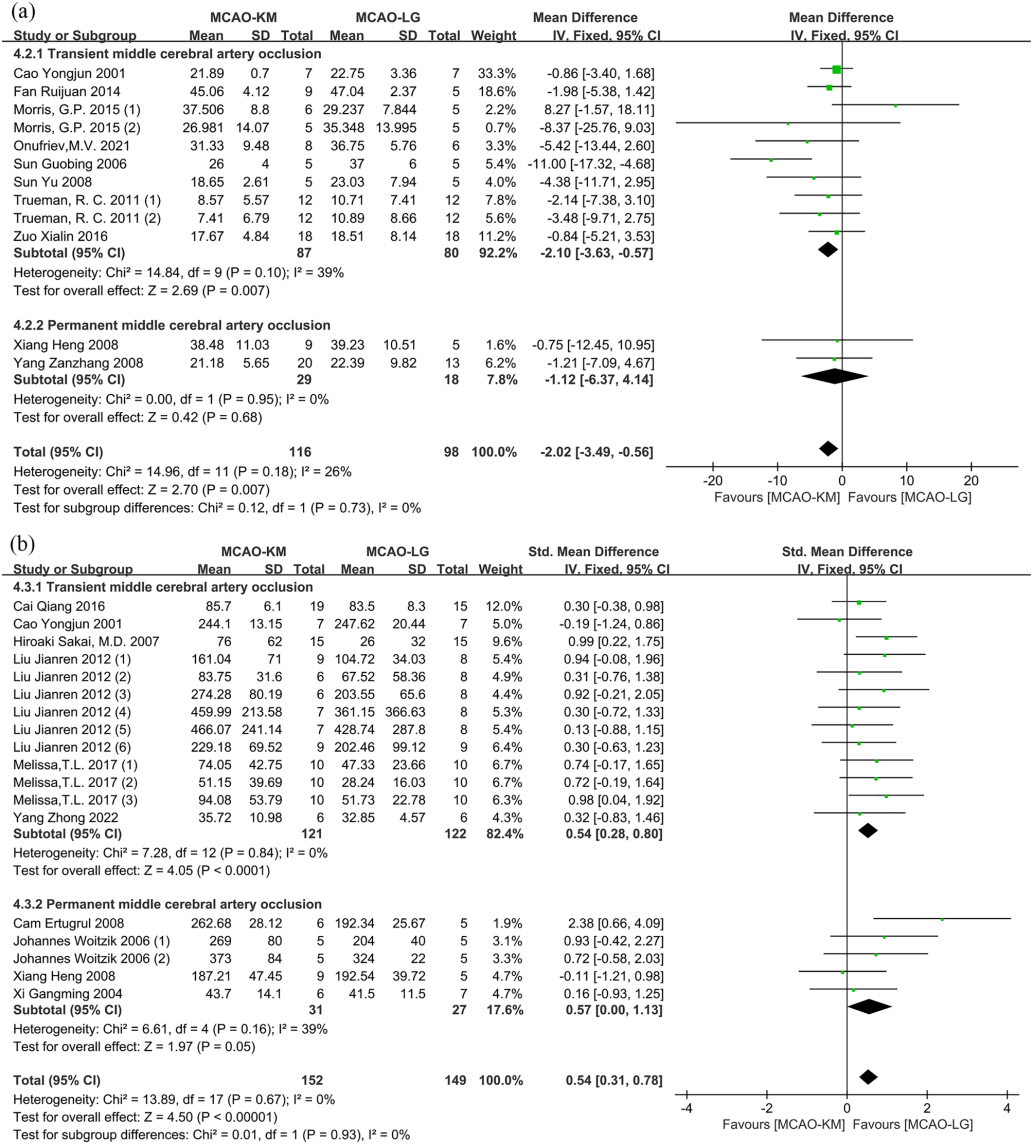
Comparison of cerebral infarction size between MCAO-KM and MCAO-LG split by (**a**) infarction rate and (**b**) lesion volume.

Eleven studies^12,14,15,21,24,26–28,37–39^ evaluated the cerebral infarction rates between two modeling methods (Table 4), and one study^27^ was excluded due to lack of sample size. Overall, the cerebral infarction rates of the MCAO-KM were markedly lower than the MCAO-LG (MD = − 3.37; 95% CI [− 4.71, − 2.04], *p* < 0.00001; heterogeneity *I*^2^ = 82%, *p* < 0.00001). And sub-analyses suggest that the MCAO-KM gets low cerebral infarction rates in the tMCAO subgroup, but not in the pMCAO subgroup (MD = − 3.53, 95% CI [− 4.91, − 2.15], *p* < 0.00001, heterogeneity *I*^2^ = 84%, *p* < 0.00001; and MD = − 1.12; 95% CI [− 6.37, 4.14]; *p* = 0.68 heterogeneity *I*^2^ = 0%, *p* = 0.95, respectively) (Supplementary Fig. 5a). After the removal of one study^24^, the distinction of cerebral infarction rates remained significant between the two methods, and the heterogeneity of the overall effect as well as the tMCAO subgroup was eliminated (SMD = − 2.10, 95% CI [− 3.63, − 0.57], *p* = 0.007, heterogeneity *I*^2^ = 39%, *p* = 0.10 in tMCAO subgroup; and SMD = − 2.02, 95% CI [− 3.49, − 0.56], *p* = 0.007, heterogeneity *I*^2^ = 26%, *p* = 0.18 in overall effect) (Fig. 9a). The *p*-value of Egger's regression test in the tMCAO subgroup was 0.390, verifying no significant publication bias (Supplementary Fig. 5b,c).

**Table 4:** Detailed table of cerebral infarction rates test.

Lead author	Year	Test method	Ischemia duration	Reperfusion duration
Cao Yongjun^37^	2001	TTC staining	6 h	18 h
Fan Ruijuan^14^	2014	TTC staining	20 min	1 day
Morris, G.P.^15^ (thin filament)	2015	TTC staining	1 h	4 h
Morris, G.P.^15^ (thick filament)	2015	TTC staining	1 h	4 h
Onufriev, M.V.^28^	2021	TTC staining	1 h	3 days
Smith, H.K.^24^ (1)	2015	TTC staining	0.5 h	1 day
Smith, H.K.^24^ (2)	2015	TTC staining	0.5 h	7 days
Sun Guobing^38^	2006	TTC staining	2 h	1 day
Sun Yu^12^	2008	TTC staining	2 h	1 day
Trueman, R. C.^22^	2011	Magnetic resonance imaging	1 h	1 day
Trueman, R. C.^22^	2011	Magnetic resonance imaging	1 h	1 day
Wang Dongliang^27^	2019	TTC staining	1 h	1 day
Zuo Xialin^26^	2016	TTC staining	1 h	1 day
Xiang Heng^20^	2008	TTC staining	Permanent	7 days
Yang Zanzhang^21^	2008	TTC staining	Permanent	1 day

Twelve studies^13,18–20,23,25,30,31,35–37,40^ evaluated the distinction of cerebral lesion volumes between the two modeling methods (Table 5). Meta-analysis suggests that the average cerebral lesion volumes of the MCAO-KM rodents were higher than the MCAO-LG rodents (SMD = 0.68; 95% CI [0.46, 0.91], *p* < 0.00001; heterogeneity *I*^2^ = 59%, *p* = 0.003). And sub-analyses suggest that the MCAO-KM animals show a higher average cerebral infarction volume than MCAO-LG in both tMCAO and pMCAO subgroups (SMD = 0.61; 95% CI [0.36, 0.86], *p* < 0.00001; heterogeneity *I*^2^ = 57%, *p* = 0.003; and SMD = 0.96, 95% CI [0.47, 1.45], *p* = 0.0001, heterogeneity *I*^2^ = 66%, *p* = 0.001, respectively) (Supplementary Fig. 6a). Sensitivity analyses pointed out that two studies^25,31^ should be the source of heterogeneity. After removing these studies, the heterogeneity was significantly reduced. Sub-analyses suggest that the MCAO-KM animals showed a higher average cerebral lesion volume in tMCAO but not pMCAO subgroup (SMD = 0.54; 95% CI [0.28, 0.80]; *p* < 0.0001, heterogeneity *I*^2^ = 0%, *p* = 0.84 in tMCAO; SMD = 0.57, 95%CI [0.00, 1.13], *p* = 0.05, heterogeneity *I*^2^ = 39%, *p* = 0.16 in pMCAO; and SMD = 0.54, 95%CI [0.31, 0.78], *p* < 0.00001, heterogeneity *I*^2^ = 0%, *p* = 0.67 in overall effect) (Fig. 9b). The *p*-value of Egger's regression test in the tMCAO subgroup was 0.679, indicating no significant publication bias (Supplementary Fig. 6b,c).

**Table 5:** Detailed table of cerebral infarction volume test.

Lead author	Year	Test method	Ischemia duration	Reperfusion duration
Cai Qiang^13^	2016	TTC staining	1 h	7 days
Cao Yongjun^37^	2001	TTC staining	6 h	18 h
Helena Justic^31^	2022	Magnetic resonance imaging	0.5 h	2 days
Hiroaki Sakai^40^	2007	Succinate dehydrogenase staining	50 min	14 days
Liu Jianren (1)^23^	2012	T2-weighted MRI	1.5 h	1 day
Liu Jianren (2)^23^	2012	Diffusion weighted imaging	1.5 h	1 h
Liu Jianren (3)^23^	2012	Diffusion weighted imaging	1.5 h	1 day
Liu Jianren (4)^23^	2012	Perfusion-weighted imaging	1.5 h	1 h
Liu Jianren (5)^23^	2012	Perfusion-weighted imaging	1.5 h	1 day
Liu Jianren (6)^23^	2012	TTC staining	1.5 h	1 day
Melissa, T.L. (1)^36^	2017	T2-weighted MRI	1 h	2 days
Melissa, T.L. (2)^36^	2017	Diffusion weighted imaging	1 h	2 days
Melissa, T.L. (3)^36^	2017	TTC staining	1 h	2 days
Yang Zhong^30^	2022	TTC staining	1.5 h	28 days
Zheng Jianfeng (1)^25^	2016	TTC staining	2 h	1 day
Cam Ertugrul^19^	2008	Toluidine blue staining	Permanent	1 day
Johannes Woitzik (1)^35^	2006	TTC staining	Permanent	8 h
Johannes Woitzik (2)^35^	2006	TTC staining	Permanent	1 day
Xiang Heng^20^	2008	TTC staining	Permanent	7 days
Xi Gangming^18^	2004	TTC staining	Permanent	7 days
Zheng Jianfeng (2)^25^	2016	TTC staining	Permanent	1 day

#### Distinction on brain edema

**Figure 10:**
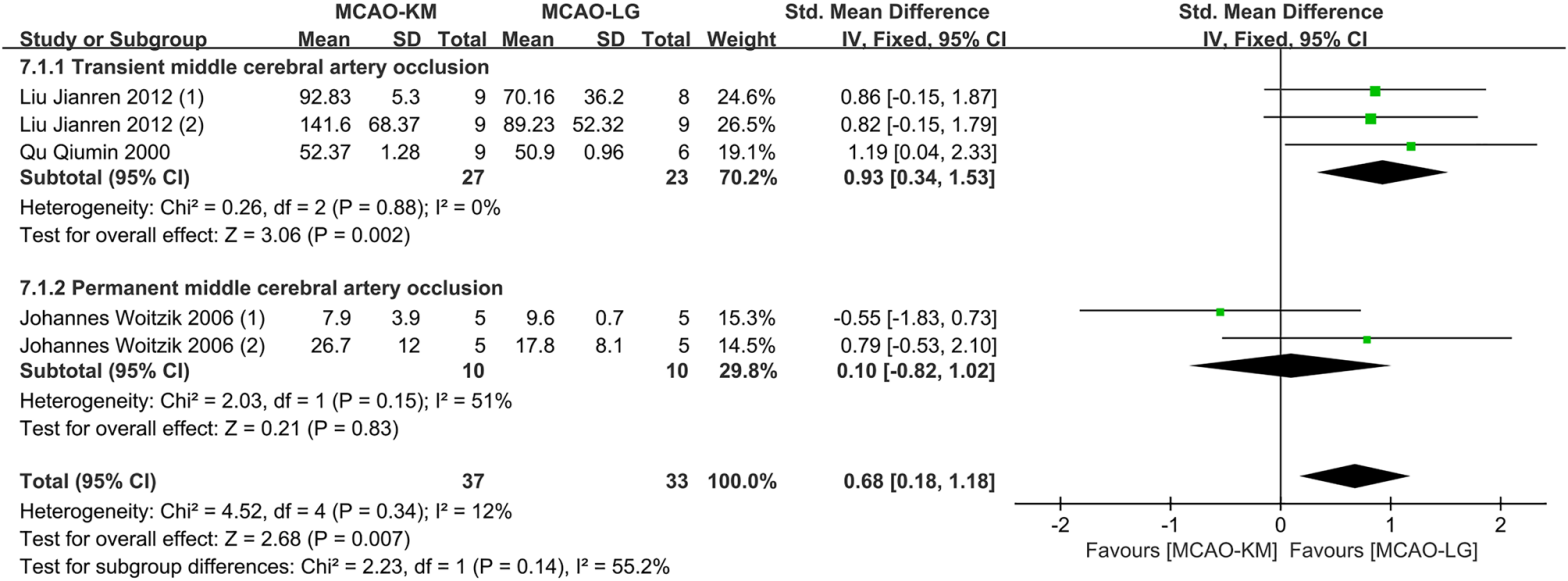
Comparison of brain edema in rodents between MCAO-KM and MCAO-LG.

The assessments of the brain edema rates were performed in six studies^23,27,31,33–35^, and two studies^27,33^ were excluded due to a lack of detailed data (Table 6). Overall, the MCAO-KM animals produced severer brain edema compared to the MCAO-LG with marked heterogeneity (SMD = 0.53, 95% CI [0.03, 1.02], *p* = 0.04, heterogeneity *I*^2^ = 71%, *p* = 0.005) (Supplementary Fig. 7). Subgroup analysis indicates that significant differences occur in the tMCAO but not in the pMCAO subgroup (SMD = 0.70, 95% CI [0.12, 1.28], *p* = 0.02, heterogeneity *I*^2^ = 78%, *p* = 0.003, and SMD = 0.10, 95% CI [− 0.82, 1.02], *p* = 0.83, heterogeneity *I*^2^ = 51%, *p* = 0.15, respectively). Sensitivity analysis uncovered that one study^31^ is a potential source of heterogeneity. After the removal of this study, the pooled estimation of brain edema between the two MCAO animals remained similar and the heterogeneity was eliminated (SMD = 0.93, 95% CI [0.34, 1.53], *p* = 0.002, heterogeneity *I*^2^ = 0%, *p* = 0.88 in tMCAO subgroup; SMD = 0.10, 95% CI [− 0.82, 1.02], *p* = 0.83, heterogeneity *I*^2^ = 51%, *p* = 0.15 in pMCAO subgroup; and SMD = 0.68, 95% CI [0.18, 1.18], *p* = 0.007, heterogeneity *I*^2^ = 12%, *p* = 0.34 in overall effect) (Fig. 10).

**Table 6:** Detailed table of brain edema test.

Lead author	Year	Ischemia duration	Reperfusion duration	Test method
Liu Jianren (1)^23^	2012	1.5 h	1 day	T2-weighted MRI
Liu Jianren (2)^23^	2012	1.5 h	1 day	TTC staining
Qu Qiumin^34^	2000	2 h	2 days	TTC staining
Johannes Woitzik (1)^35^	2006	Permanent	8 h	TTC staining
Johannes Woitzik (2)^35^	2006	Permanent	1 day	TTC staining
Helena Justic^31^	2022	0.5 h	2 days	Magnetic resonance imaging

#### Distinctions on neurological deficit scores

**Figure 11:**
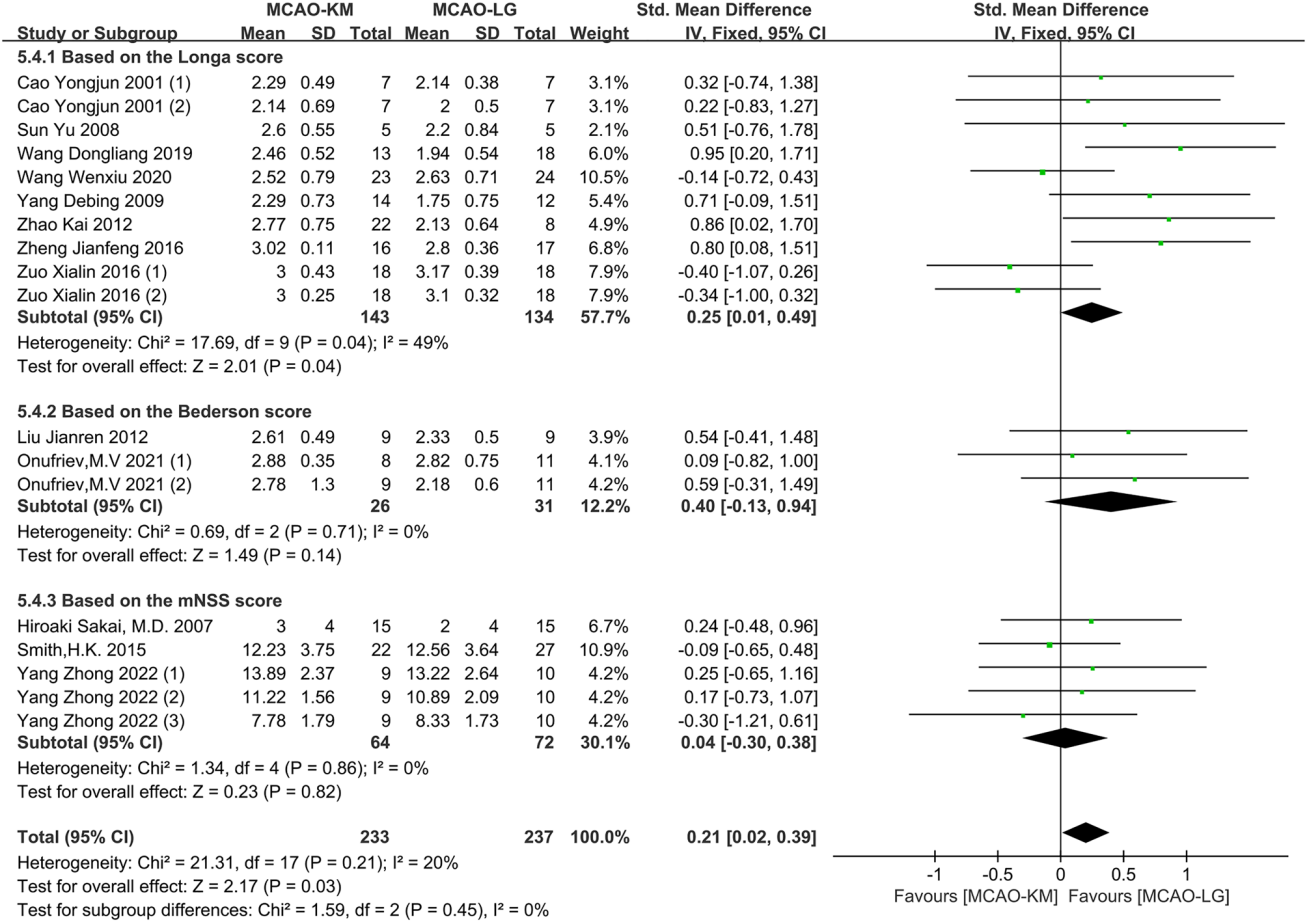
Comparison of neurological deficit score between MCAO-KM and MCAO-L.

Nineteen studies evaluated neurological deficits via Zea Longa score^12,13,17,25–27,29,32,33,35,37^, Bederson score^23,28^, modified neurological severity scores (mNSS) score^24,30,40^, and Garcia score^14,23,31^ (Table 7). Among them, three articles^13,33,35^ were excluded due to the absence of available data. Of note, the higher the the Garcia score, the better the neurobehavior, which is contrary to other scoring methods, thus, these studies using Garcia score were not pooled into the sub-analysis (Supplementary Fig. 8). Overall, the average neurological deficit scores of the MCAO-KM animals were significantly higher than that of MCAO-LG animals without heterogeneity (SMD = 0.21, 95% CI [0.02, 0.39], *p* = 0.03, heterogeneity *I*^2^ = 20%, *p* = 0.21) **(**Fig. 11).

**Table 7:** Detailed table of neurological deficit scores.

Lead author	Year	Ischemia duration	Reperfusion duration	Scoring method
Cao Yongjun (1)^37^	2001	6 h	6 h	Zea Longa
Cao Yongjun (2)^37^	2001	6 h	18 h	Zea Longa
Sun Yu^12^	2008	2 h	1 day	Zea Longa
Wang Dongliang^27^	2019	1 h	1 day	Zea Longa
Wang Wenxiu^29^	2020	2 h	2 h	Zea Longa
Yang Debing^17^	2009	2 h	1 day	Zea Longa
Zhao Kai^32^	2012	100 min	4 h	Zea Longa
Zheng Jianfeng (1)^25^	2016	2 h	1 day	Zea Longa
Zheng Jianfeng (2)^25^	2016	Permanent	1 day	Zea Longa
Zuo Xialin (1)^26^	2016	1 h	6 h	Zea Longa
Zuo Xialin (2)^26^	2016	1 h	1 day	Zea Longa
Hiroaki Sakai^40^	2007	50 min	14 days	mNSS
Smith, H.K. (1)^24^	2015	0.5 h	1 day	mMSS
Smith, H.K. (2)^24^	2015	0.5 h	2 days	mMSS
Yang Zhong (1)^30^	2022	1.5 h	1 day	mNSS
Yang Zhong (2)^30^	2022	1.5 h	7 days	mNSS
Yang Zhong (3)^30^	2022	1.5 h	28 days	mNSS
Liu Jianren^23^	2012	1.5 h	1 day	Bederson
Onufriev, M. V (1)^28^	2021	1 h	1 day	Bederson
Onufriev, M. V (2)^28^	2021	1 h	3 days	Bederson
Helena Justic (1)^31^	2022	0.5 h	2 days	Garcia
Helena Justic (2)^31^	2022	0.5 h	9 days	Garcia
Liu Jianren^23^	2012	1.5 h	1 day	Garcia
Fan Ruijuan (1)^14^	2014	No report	1 day	Garcia
Fan Ruijuan (2)^14^	2014	No report	1 day	Garcia

## Discussion

The MCAO-KM and MCAO-LG modeling methods are the two most common ways of achieving MCAO as classical surgical approaches in preclinical experiments, and they are invaluable for researchers to understand stroke patho-physiology and develop new therapeutics. However, in preclinical studies, the two classic intraluminal filament approaches for MCAO are considered alternatives, and some researchers even believed that they are interchangeable^23,26^. Therefore, it is critical to distinguish between them to develop an appropriate stroke model.

### Distinctions in model establishment

The fundamental distinction between the MCAO-KM and MCAO-LG methods is the surgical procedure's complexity. Because most anesthetics have neuroprotective benefits against ischemic stroke, the prolonged duration of anesthesia may confuse the therapeutic effect of candidate medications^41^. According to our experience, the MCAO-KM is easier to perform because it does not require ligating the ECA and its embranchment, which shortens operation time (Fig. 4a) and may decrease eating difficulties caused by injuries of soft tissues and cranial nerves^42^. Furthermore, CCA is easier to plug a filament than ECA because the former is thicker and straighter than the latter, which explains the reason why MCAO-KM shows a higher success rate of insertion filament and shorter operation time than MCAO-LG.

SAH is one of the leading causes of postoperative mortality in the intraluminal filament MCAO model, usually caused by excessive insertion of the filament^43^. Generally, the optimal insertion depth depends on the species, and the propulsion of the filament stops when the finger feels a slight resistance after entering the intracranial cavity^13,24^. Arterioles derive from the ECA are prone to bleeding, thus, the ligature around ECA need to be tied more tightly (Fig. 1), which results in a reduced perception of finger protraction resistance. It may be a direct factor causing elevated SAH in MCAO-LG animals with a higher risk of intracranial vascular puncture (Fig. 6).

Low mortality is advantageous, representing an improved model of MCAO, allowing a reduced number of rodents. There was no overall difference in model mortality between the two surgical methods, but distinct results were found in the subgroup analyses (Fig. 5). In the tMCAO subgroup, the animal mortality of the MCAO-KM was higher than MCAO-LG, which may be related to the long-term hypoperfusion of the ischemic hemisphere caused by permanent ligation of CCA^24^. Conversely, in the pMCAO subgroup, higher mortality occurs in MCAO-LG, which may be interpreted by the greater surgical injury caused by a relatively complex procedure. Therefore, in the preparation of pMCAO model, inserting suture from CCA is a more sensible choice.

Model success rate, as a comprehensive index, reflects the difficulty of operation. For the tMCAO model, there is no significant difference between the two methods, however, for the pMCAO model, the success rate of MCAO-KM is higher than MCAO-LG (Fig. 7). Given the foregoing, it is reasonable to believe that the MCAO-KM method is simple to model establishment. In other words, the MCAO-LG method is challenging for a fledgling.

### Distinctions in CBF

The routine and extent of reperfusion are the main distinctions between the two methods in patho-physiology. In short, the MCAO-LG animals' reperfusion is mainly derived from bilateral CCA, whereas the MCAO-KM animals' reperfusion is primarily derived from contralateral CCA via the circle of Willis because the ipsilateral CCA was permanently ligated (Fig. 1). After plugging in the filament, CBF detection revealed no significant difference between the two groups (Fig. 8a), and the decline was all about 60% of the pre-ischemia level^19,35^. Interestingly, reperfusion in MCAO-KM animals reached only 50% of baseline levels, while, in MCAO-LG rodents, reperfusion could rapidly restore to near-normal levels **(**Fig. 8b). Furthermore, high-resolution MR angiography defines the MCAO-KM as an ischemia–reperfusion model with chronic hypoperfusion, whereas the MCAO-LG method achieves ischemia complete reperfusion^31^.

Surge reperfusion observed with the removal of the filament may be like that observed with clinical endovascular thrombectomy^44^. Considering the insufficient reperfusion of MCAO-KM animals, some scholars believe that the MCAO-LG method with complete reperfusion should be given priority in the exploration of the neuroprotective agents on cerebral ischemia–reperfusion injury^44^. However, only 7–10% of patients in developed countries receive effective thrombolytic therapy within the therapeutic time window^45,46^. What's worse, stroke patients—approximately 30.9–72.3%—do not achieve full revascularization despite receiving thrombolytic therapy^47–49^. Besides, the researchers observed that the brain would provide hypoperfusion blood to the infarcted core via collateral vessels in humans or rodents that did not receive recanalization treatment^50,51^. Thus, stroke patients with chronic hypoperfusion injury were more common than that with complete reperfusion^52^. In other words, although the ischemia stroke model prepared by the MCAO-KM method does not achieve complete reperfusion, it may be close to the main situation of no or incomplete thrombolysis.

### Distinctions in cerebral infarction size, brain edema rate and neurological deficit score

We evaluated the infarction size in the forms of volume and percentage, interestingly, the results from the two forms were opposites in the tMCAO model, and little difference was observed in the pMCAO model (Fig. 9). As is well known, full ischemia–reperfusion rapidly mobilizes amounts of erythrocytes and hemoglobin^53^, and mitochondrial dysfunction causes the oxygen transported by this hemoglobin to be converted into amounts of reactive oxygen species^54,55^. Additionally, via interacting with endothelial cells, a considerable number of circulating immune cells enter the brain parenchyma^56^. All of the aforementioned factors lead to untimely oxidative stress and inflammatory reactions, which result in secondary CIRI. Another investigation discovered that rats that received quick flow restoration had more infarctions than those who underwent incremental flow restoration^57^. Thus, it is speculated that the bigger cerebral infarction in the MCAO-LG rodents is caused by the more severe CIRI produced by the remarkable recovery of CBF.

Ligation of a unilateral CCA significantly destroys the blood–brain barrier and increases brain water content in rats^58^, Similar to this, individuals who have failed recanalization experience an increase and expansion of the edema volume^59^. Therefore, it may be reasonable to assume that the permanent ligature of CCA explains why the MCAO-KM technique results in more severe brain edema (Fig. 10). In addition, giving that edema-corrected infarction progression is greater than the edema progression, the level of edema is insufficient to account for the overall expansion of infarction volume^59^. Thus, considering the peak of cerebral edema, a modified formula rather than pure infarction volume should be used when estimating infarct size^60^.

Neurological abnormalities and cognitive deterioration are linked to chronic cerebral hypoperfusion. In this study, we discovered that neurological impairments in MCAO-KM rodents were more severe owing to the protracted hypoperfusion brought on by unilateral CCA ligation (Fig. 11). Important to keep in mind is that the impact of CCA permanent ligature on cognitive function may negate the effectiveness of emerging treatment options that aim to treat stroke-induced vascular dementia or stroke with Alzheimer's disease^30^.

### Distinctions in inflammation

The development of ischemia stroke is heavily influenced by inflammation, and the inflammatory response brought on by various surgical techniques is time-dependent. As early as 4–6 h after surgery, neutrophils were observed in the ischemic core following ischemia^61,62^. The interaction between leukocytes and endothelial cells was studied by Smith et al.^24^, who discovered that both animals' ischemic brain tissue was filled with a significant number of rolling leukocytes. Interestingly, cells remaining stationary within the vessel only occurred in MCAO-LG after urgent reperfusion. Another study^27^ discovered that NF-κB expression in the ischemic core was considerably greater in the MCAO-LG than the MCAO-KM after 24 h of reperfusion. However, during the subacute stage of ischemic stroke, Onufriev et al.^28^ discovered that IL-1 together with corticosterone discharge and amass in the MCAO-KM rat hippocampus, indicating that MCAO-KM predisposes the animals to corticosterone-dependent distant neuroinflammatory hippocampal injury. Furthermore, in terms of long-term inflammatory responses, Yang et al.^30^ found no significant difference between the two modeling approaches in astrocyte and microglial activation, as well as apoptotic neuronal death. Thus, MCAO-LG may have a greater inflammatory response in the acute phase, whereas MCAO-KM may have a stronger inflammatory response in the subacute period.

### Distinctions in other aspects

Other subtle differences exist between the two modeling methods, and these discrepancies are important considerations in fundamental research. For example, mice's pterygopalatine artery, the first internal carotid artery branch, is the source of the ocular artery^31^. The majority of retinal reactions to ischemia coincide with brain tissue in MCAO-KM animals, with substantial atrophy of the inner retinal layers due to permanent CCA ligation, whereas MCAO-LG mice revealed no serious histological retinal abnormalities. Thus, the MCAO-KM is considered to be a better way to study the patho-physiology of cerebral combined retinal ischemia^31^.

Additionally, equally important is whether the occluding suture covers the posterior communicating artery (pCom). The heat-blunted tip of Zea Longa likely avoids the pCom, but the silicone coating of Koizumi may and frequently does occlude the pCom, particularly in mice^35^. In this study, we observed that the CBF of MCAO-KM is less than that of MCAO-LG during ischemia although there was no significant difference (Fig. 7a), or that pCom obstruction had no significant effect on CBF. The reason for this may be related to the detected area and the detection time between different studies (Table 3). It may also be that most studies used the same filament for controlling variables (Table 1). In any case, the processing of the filament tip cannot be ignored before model establishment, which is one of the inspirations brought by the two molding methods.

### Limitations and outlooks

Even though MCAO-LG and MCAO-KM are extensively utilized across the world, high-quality publications dedicated to a direct comparison of the two approaches are still limited, which is why just 28 pieces of literature were considered in this study. Aside from the vessel insertion position, the processing and insertion depth of the filament may also lead to differences in modeling establishment and brain injury, which may explain the potential heterogeneity of some mete-analysis results. Therefore, more related research is needed to solve this issue, and given the current developments in molecular biotechnology, it is crucial to identify the disparities in molecular biological mechanisms to develop effective therapies for ischemic stroke.

## Conclusion

Based on the meta-analysis results, we conclude that the surgical procedure—MCAO-KM or MCAO-LG—should not be chosen arbitrarily, and the filament insertion route has a substantial effect on the model establishment, CBF, and CIRI.

In summary, MCAO-KM has advantages in the model establishment, such as shorter operation time, easier filament insertion, lower risk of SAH, and higher model success rate, it is more appropriate for the study of ischemia-induced brain edema due to chronic hypo-reperfusion with a higher risk of postoperative death and severe neurological deficits. Whereas MCAO-LG is better suited for the study of acute CIRI due to its ability to preserve CCA integrity with a sophisticated and challenging surgical procedure, it has low model mortality, high CBF replenishment, large cerebral infarction, and an acute inflammatory response.

Therefore, instead of arbitrary variables, this discovery may provide scientists with actual parameters for selecting a suitable intraluminal filament MCAO model.

## Supplementary Information

Supplementary Information.

## Data Availability

All the data are available within each publication and in our figures and table. For further inquiries regarding the data extraction, raw data included, and/or analyses, email 3192424728@qq.com.

## Abbreviations

BBB: Blood–brain barrier
CBF: Cerebral blood flow
CCA: Common carotid artery
CIRI: Cerebral ischemia–reperfusion injury
ECA: External carotid artery
ICA: Internal carotid artery
MCA: Middle cerebral artery
MCAO: Middle cerebral artery occlusion
mNSS: Modified neurological severity scores
pMCAO: Permanent middle cerebral artery occlusion
PPA: Pterygopalatine artery
SAH: Subarachnoid hemorrhage
TTC staining: 2,3,5-Triphenyl tetrazolium chloride staining
tMCAO: Transient middle cerebral artery occlusion
