# Supplementary material for: Distinctions between the Koizumi and Zea Longa methods for middle cerebral artery occlusion (MCAO) model: a systematic review and meta-analysis of rodent data
Source: Sci Rep. 2023 Jun 23;13:10247. doi: 10.1038/s41598-023-37187-w (PMC10290095; doi:10.1038/s41598-023-37187-w)
Supplement: Supplementary file 1 — Supplementary Information. [file 41598_2023_37187_MOESM1_ESM.pdf]

Supplementary material

Article title: Distinctions between the Koizumi and Longa methods for middle cerebral artery occlusion (MCAO) model: A systematic review and meta-analysis of rodent data

Journal name: Scientific Reports

Author names: Yong Li[1], Li Tan[1], Caixia Yang[1], Liying He[1], Lin Liu[1], Bowen Deng[1], Sijing Liu, Jinlin Guo[1]

Affiliation and e-mail address of the corresponding author:

Jinlin Guo (guo596@cdutcm.edu.cn),

College of Pharmacy, Chengdu University of Traditional Chinese Medicine, Chengdu, China

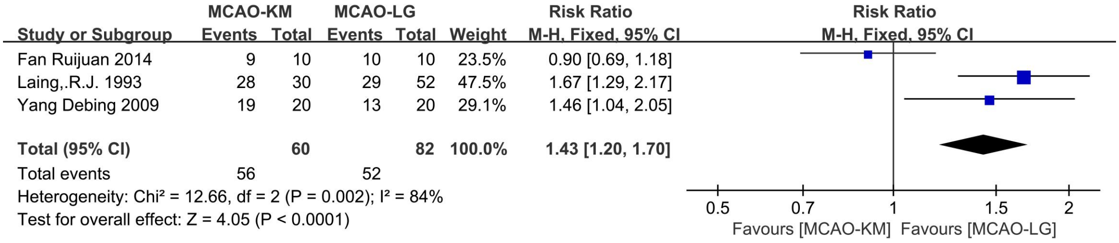

Figure 1. Forest plot of the filament insertion success rate before sensitivity analysis between the MCAO-KM and the MCAO-LG.

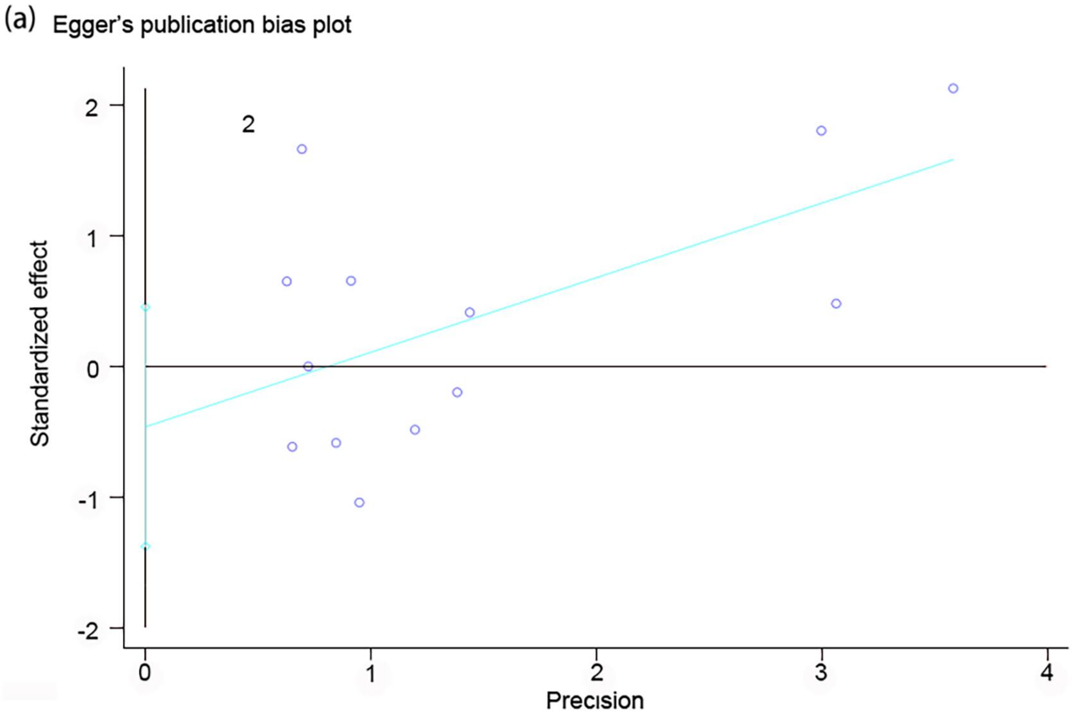

(b) Egger's test

Std_Eff	Coef.	Std. Err.	t	P>|t|	[95% Conf.	Interval]
slope	.5709336	.2349298	2.43	0.033	.0538567	1.088011
bias	-.4621282	.4166149	-1.11	0.291	-1.379091	.4548351

Figure 2. Postoperative mortality of MCAO model between the MCAO-KM and the MCAO-LG. (a) Egger's publication bias plot, and (b) Egger's test results.

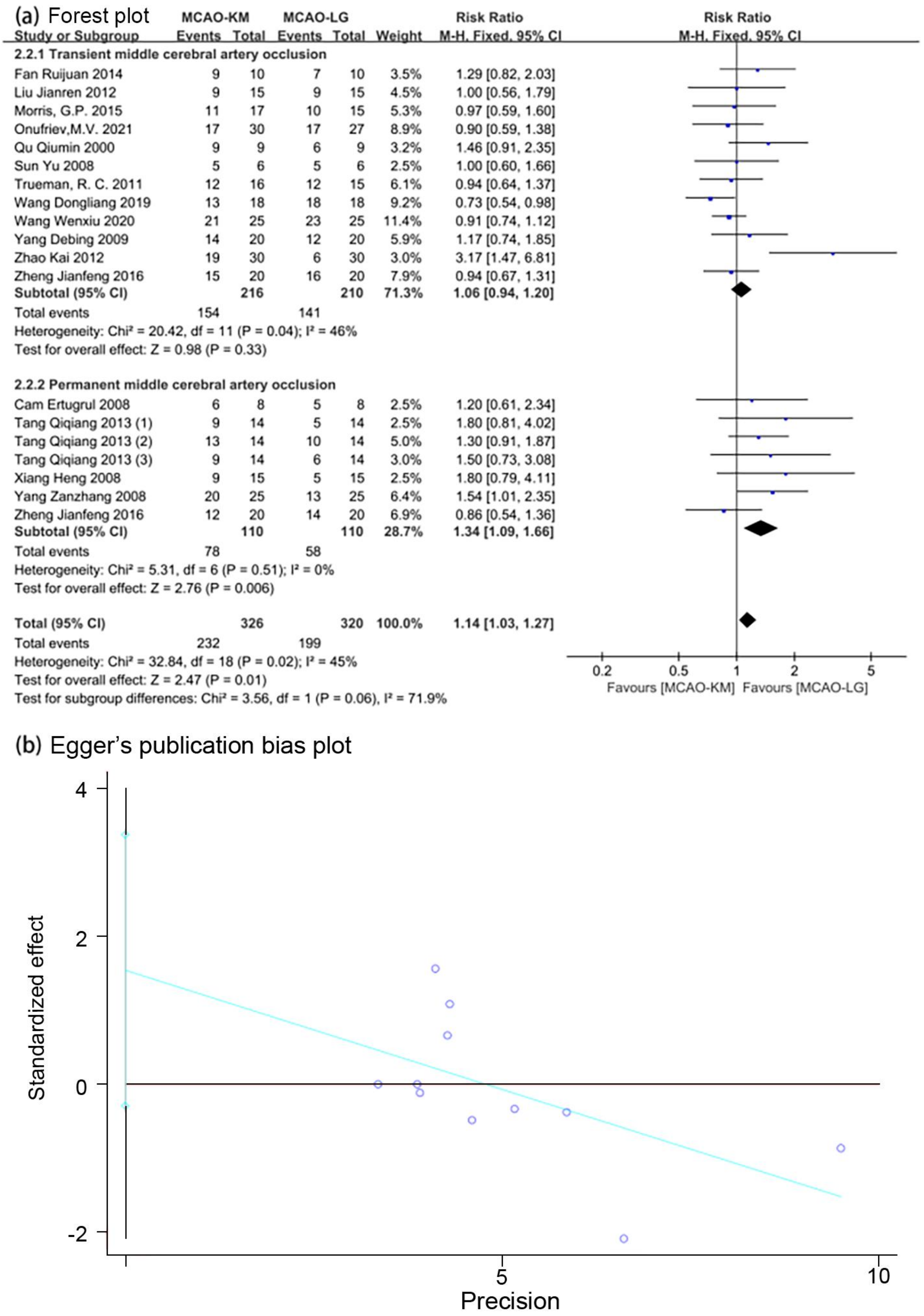

(c) Egger's test

Std_Eff	Coef.	Std. Err.	t	P>|t|	[95% Conf.	Interval]
slope	-.3219122	.1519904	-2.12	0.063	-.6657384	.021914
bias	1.538604	.8084191	1.90	0.089	-.290167	3.367375

Figure 3. Success rates of MCAO model animal between the MCAO-KM and MCAO-LG. (a) Forest plot before sensitivity analysis, (b) Egger's publication bias plot, and (c) Egger's test results.

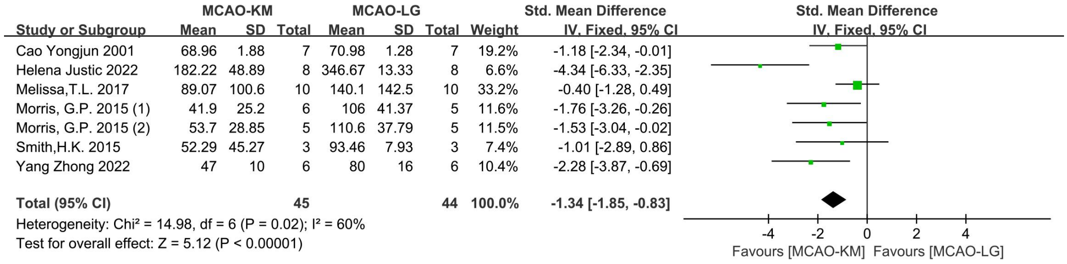

Figure 4. Forest plot of cerebral blood flow test between the MCAO-KM and the MCAO-LG.

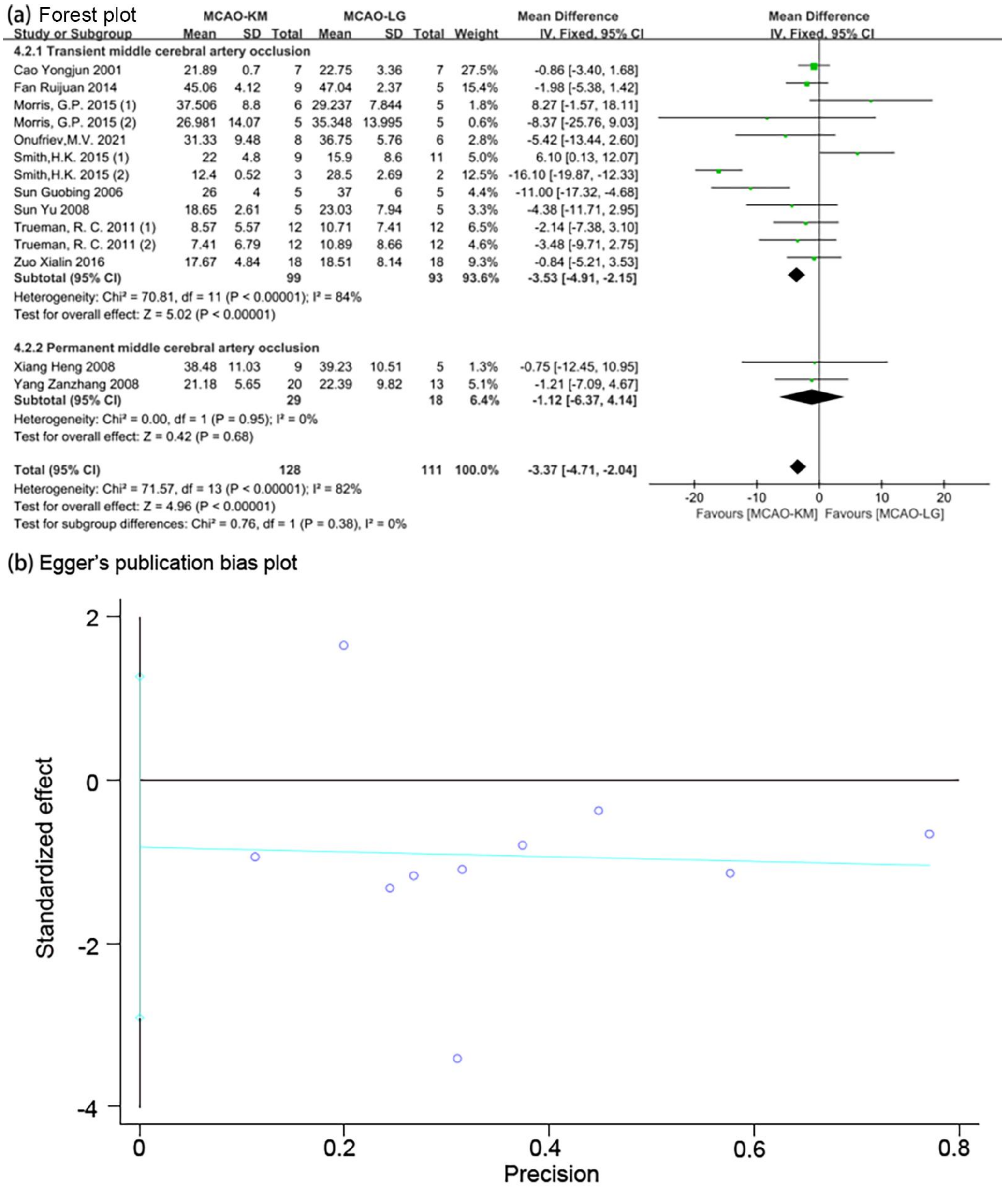

(c) Egger's test

Std_Eff	Coef.	Std. Err.	t	P>|t|	[95% Conf.	Interval]
slope	-.2895647	2.233063	-0.13	0.900	-5.439017	4.859888
bias	-.8230793	.9058966	-0.91	0.390	-2.912081	1.265922

Figure 5. Cerebral infarction rate between the MCAO-KM and the MCAO-LG. Forest plot (a) before sensitivity analysis, Egger's publication bias plot (b), and Egger's test results (c) are shown as follows.

(a) Forest plot

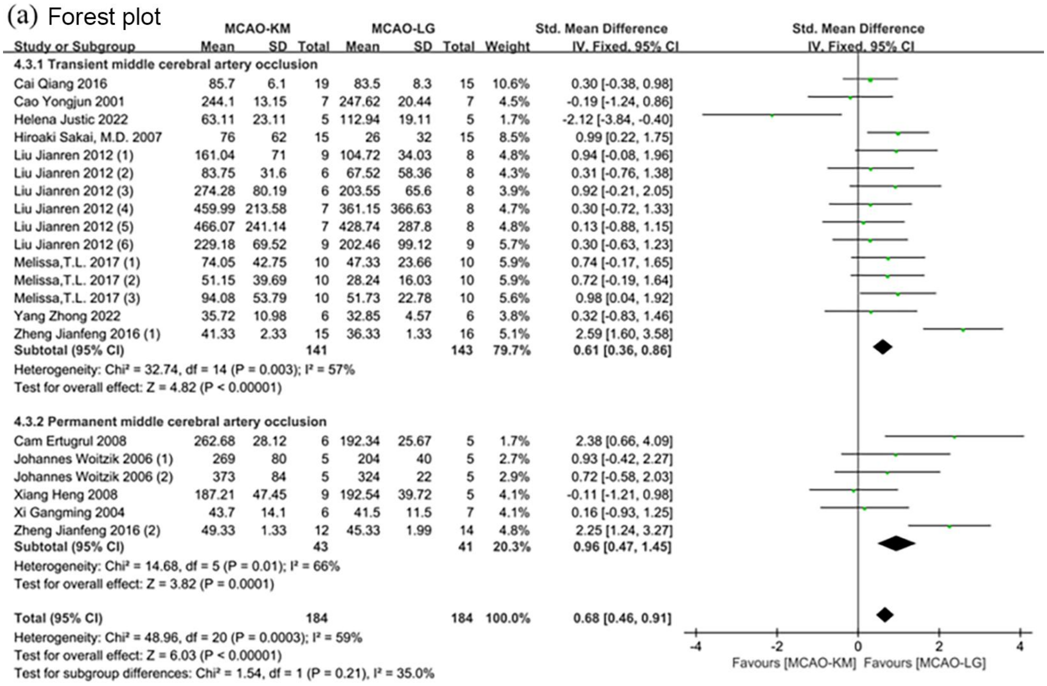

(b) Egger's publication bias plot

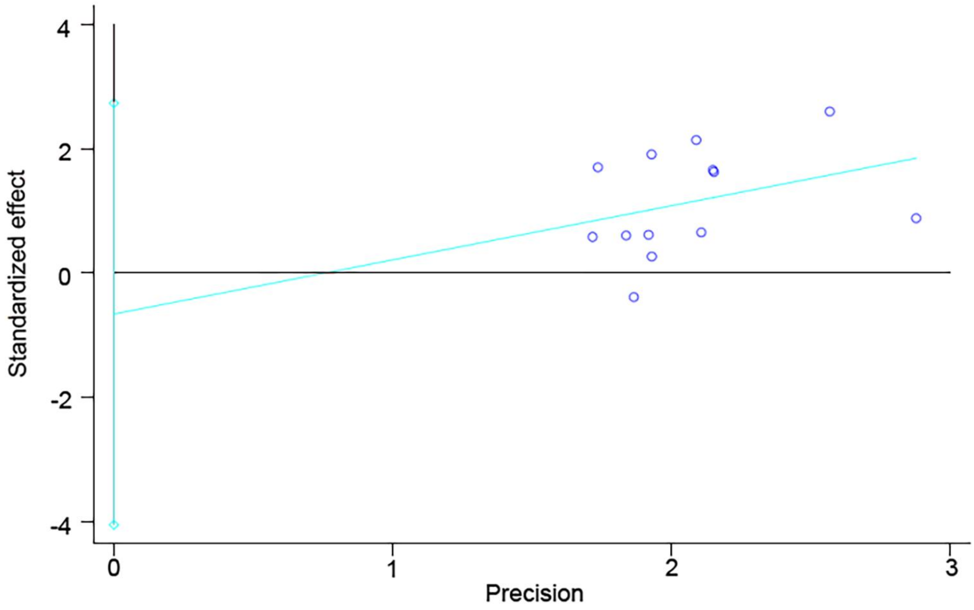

(c) Egger's test

Std_Eff	Coef.	Std. Err.	t	P>|t|	[95% Conf.	Interval]
slope	.87047	.7361794	1.18	0.262	-.74985	2.49079
bias	-.6535626	1.539623	-0.42	0.679	-4.04225	2.735125

Figure 6. Cerebral infarct volume between the MCAO-KM and the MCAO-LG. Forest plot (a) before sensitivity analysis, Egger's publication bias plot (b), and Egger's test results (c) are shown as follows.

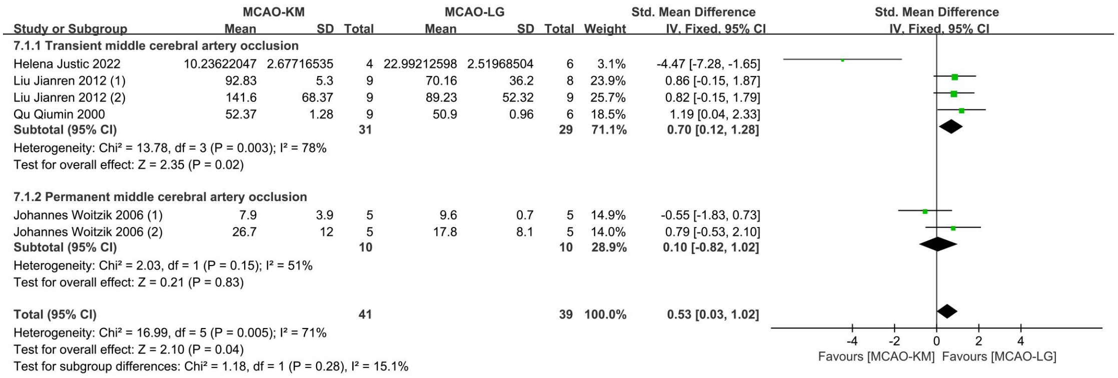

Figure 7. Forest plots of brain edema test between the MCAO-KM and the MCAO-LG.

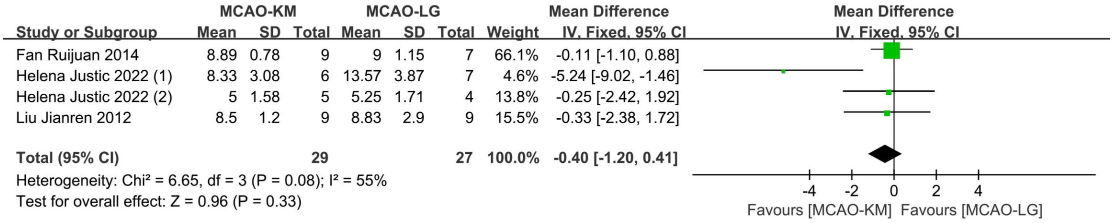

Figure 8. Garcia scores between the MCAO-KM and the MCAO-LG.
